# Supplementary figures and images for: The Mac1 ADP-ribosylhydrolase is a Therapeutic Target for SARS-CoV-2
Source: bioRxiv. 2025 Oct 6:2024.08.08.606661. Originally published 2024 Aug 9. Preprint. [Version 4] doi: 10.1101/2024.08.08.606661 (PMC11326214; doi:10.1101/2024.08.08.606661)

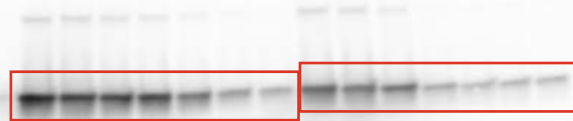

10 uM AVI-4206

DMSO

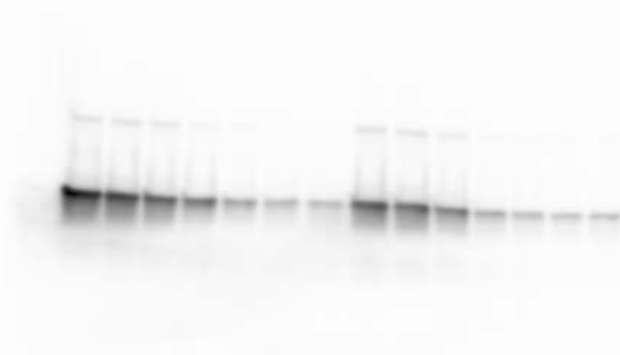

Supplement: Supplement 1 [file media-1.pdf]

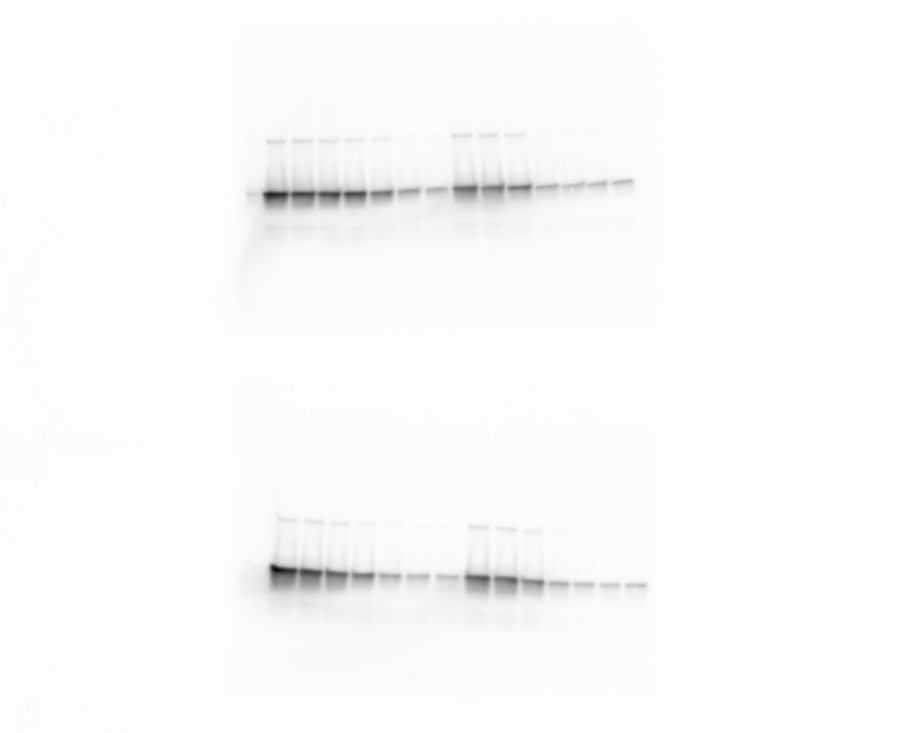

Supplement: Supplement 2 [file media-2.zip › Ashworth 2024-04-09 11h16m17s(Chemiluminescence).raw16.tif]

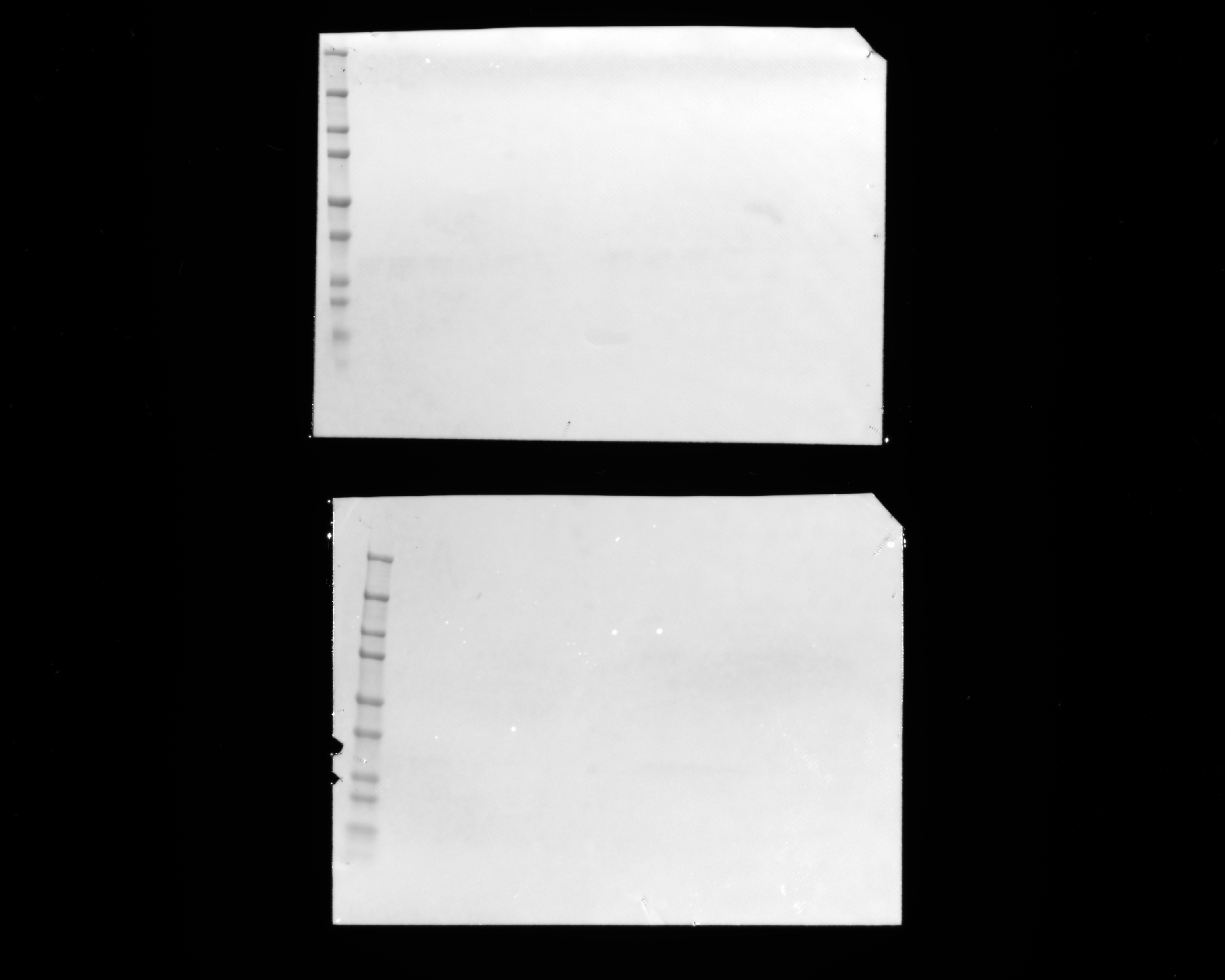

Supplement: Supplement 2 [file media-2.zip › Ashworth 2024-04-09 11h21m29s(Colorimetric).tif]

A

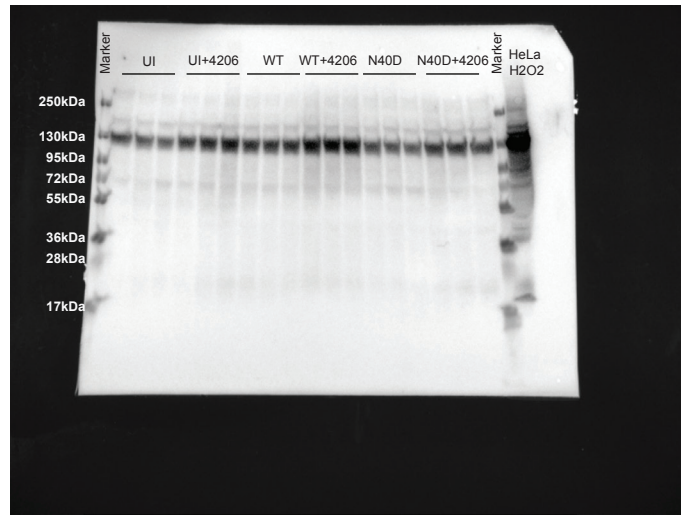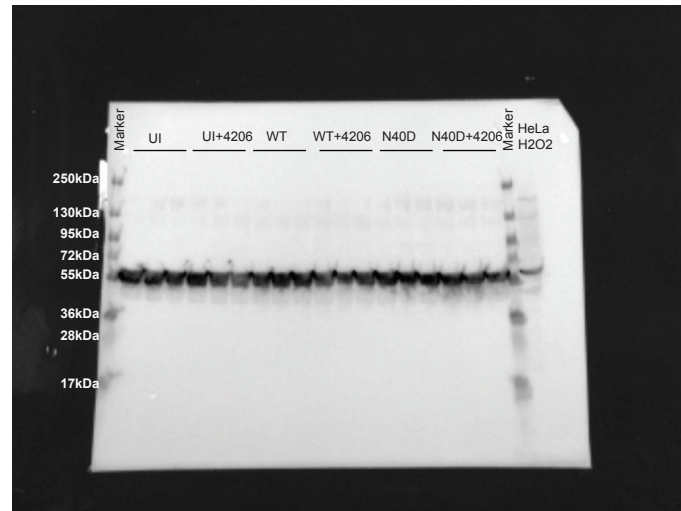

C

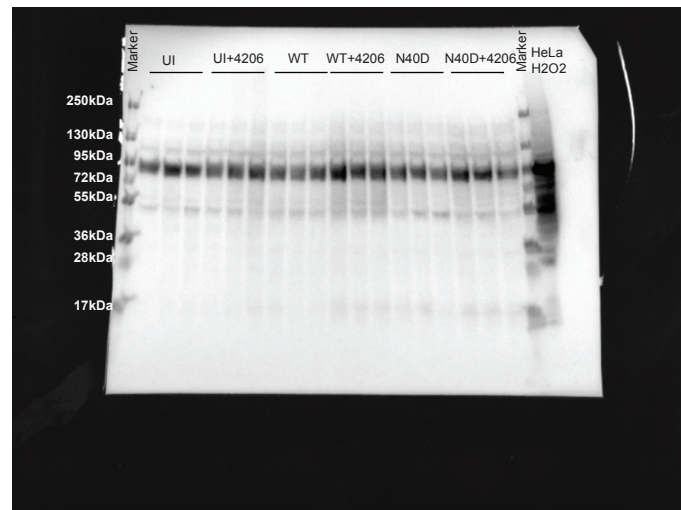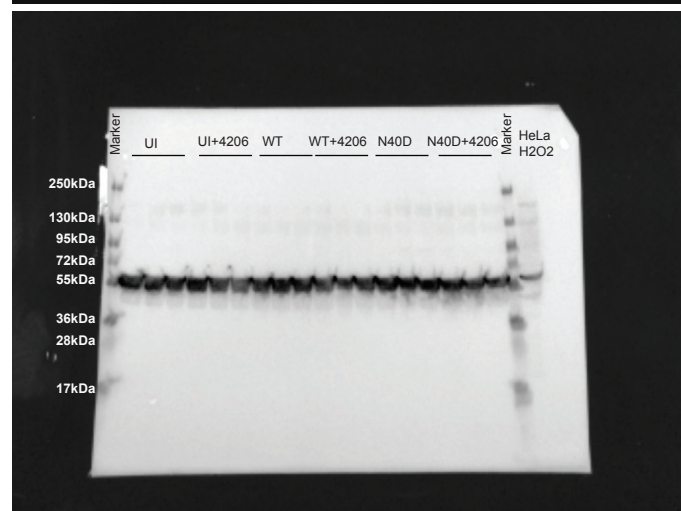

Figure 3-figure supplement 3-source data 2

Supplement: Supplement 3 [file media-3.pdf]

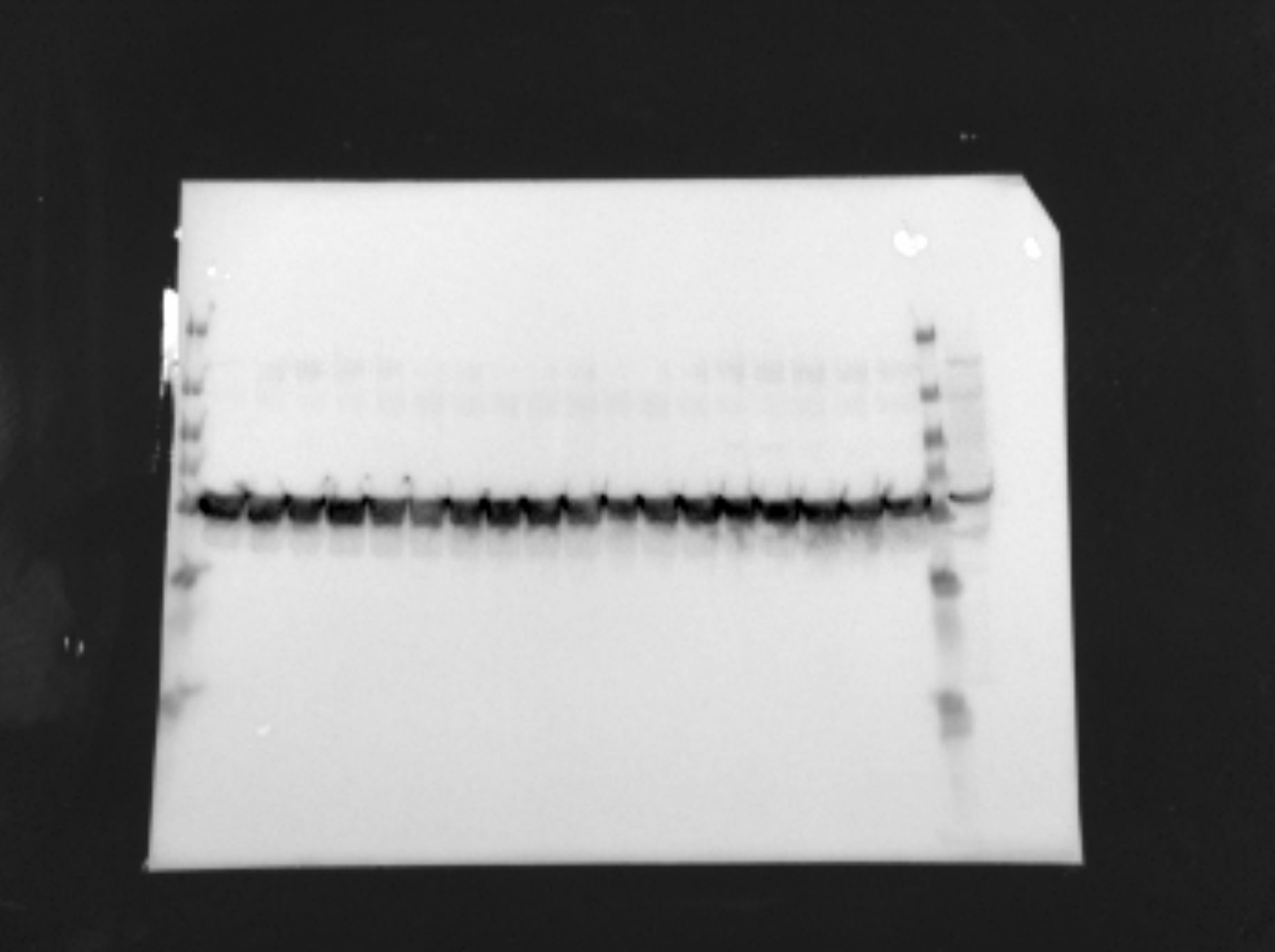

Supplement: Supplement 4 [file media-4.zip › Actin.tif]

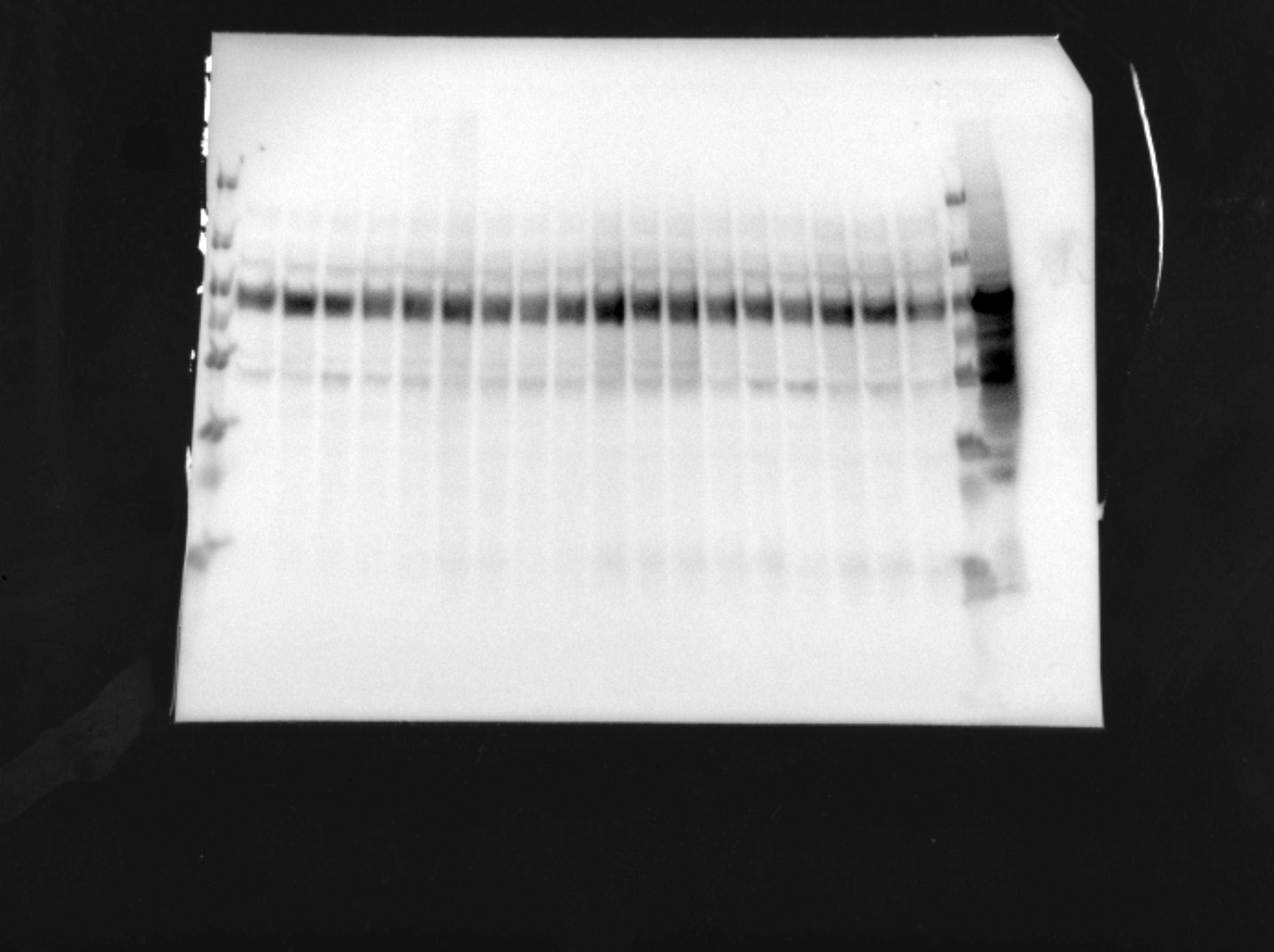

Supplement: Supplement 4 [file media-4.zip › MonoADpr.tif]

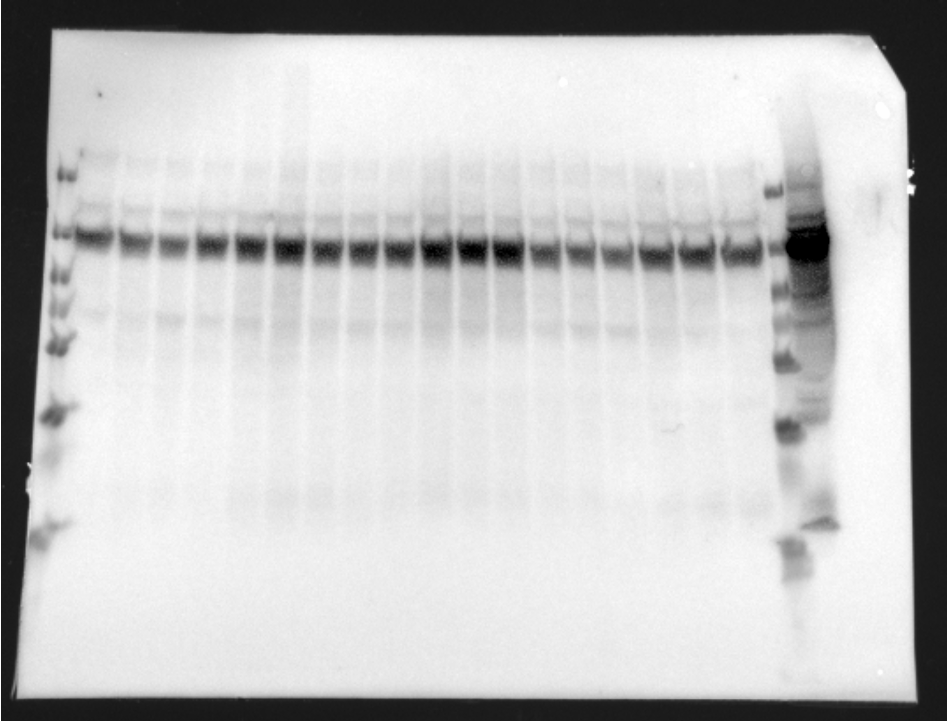

Supplement: Supplement 4 [file media-4.zip › pan-ADPr.png]
