# Supplementary material for: The Mac1 ADP-ribosylhydrolase is a Therapeutic Target for SARS-CoV-2
Source: bioRxiv. 2025 Oct 6:2024.08.08.606661. Originally published 2024 Aug 9. Preprint. [Version 4] doi: 10.1101/2024.08.08.606661 (PMC11326214; doi:10.1101/2024.08.08.606661)
Supplement: Supplement 8 [file NIHPP2024.08.08.606661v4-supplement-8.pdf]

bioRxiv preprint doi: https://doi.org/10.1101/2024.08.08.606661; this version posted October 6, 2025. The copyright holder for this preprint (which was not certified by peer review) is the author/funder. This article is a US Government work. It is not subject to copyright under 17 USC 105 and is also made available for use under a CC0 license.

Supplementary information

Supplementary File 1 - X-ray data collection and refinement deposition statistics.

Supplementary File 2 a: Eurofins scanEDGE kinase assay shows no inhibition greater than >35% at 10 µM across a panel of diverse kinases. **b:** Pharmacokinetic parameters for AVI-4206 following IV (10 mg/kg), PO (50 mg/kg), and IP (100 mg/kg) doses in male CD1 mice (n = 3 per group). **c**: ADMET panel shows no antagonist response greater than >15% at 10 µM.

Supplementary File 3 - Macrodomain protein sequences

Figure 2 - Figure Supplement 1 – Source Data 1: Labeled full gel of CETSA-WB shows thermal stabilization of FLAG-tagged Mac1 protein after treatment of A549 cells with 10 µM of AVI-4206
Figure 2 - Figure Supplement 1 – Source Data 2: Unlabeled full gels of CETSA-WB shows thermal stabilization of FLAG-tagged Mac1 protein after treatment of A549 cells with 10 µM of AVI-4206

Figure 3 - Figure Supplement 3 - source data 1: Annotated Immunoblot showing pan-ADP-ribose (panADPr) and mono-ADP-ribose (monoADPr) levels in Calu-3 cells under indicated infection conditions. UI = uninfected cells, WT = cells infected with WA1, N40D = cells infected with WA1 NSP3 Mac1 N40D, with or without 100 µM AVI-4206 treatment. HeLa cells treated with H_2O_2 were included as a positive control for the ADPr signal. Actin serves as a loading control.
Figure 3 - Figure Supplement 3 - source data 2: Full gels of immunoblot showing pan-ADP-ribose (panADPr) and mono-ADP-ribose (monoADPr) levels in Calu-3 cells under indicated infection conditions. UI = uninfected cells, WT = cells infected with WA1, N40D = cells infected with WA1 NSP3 Mac1 N40D, with or without 100 µM AVI-4206 treatment. HeLa cells treated with H_2O_2 were included as a positive control for the ADPr signal. Actin serves as a loading control.

bioRxiv preprint doi: https://doi.org/10.1101/2024.08.08.606661; this version posted October 6, 2025. The copyright holder for this preprint (which was not certified by peer review) is the author/funder. This article is a US Government work. It is not subject to copyright under 17 USC 105 and is also made available for use under a CC0 license.

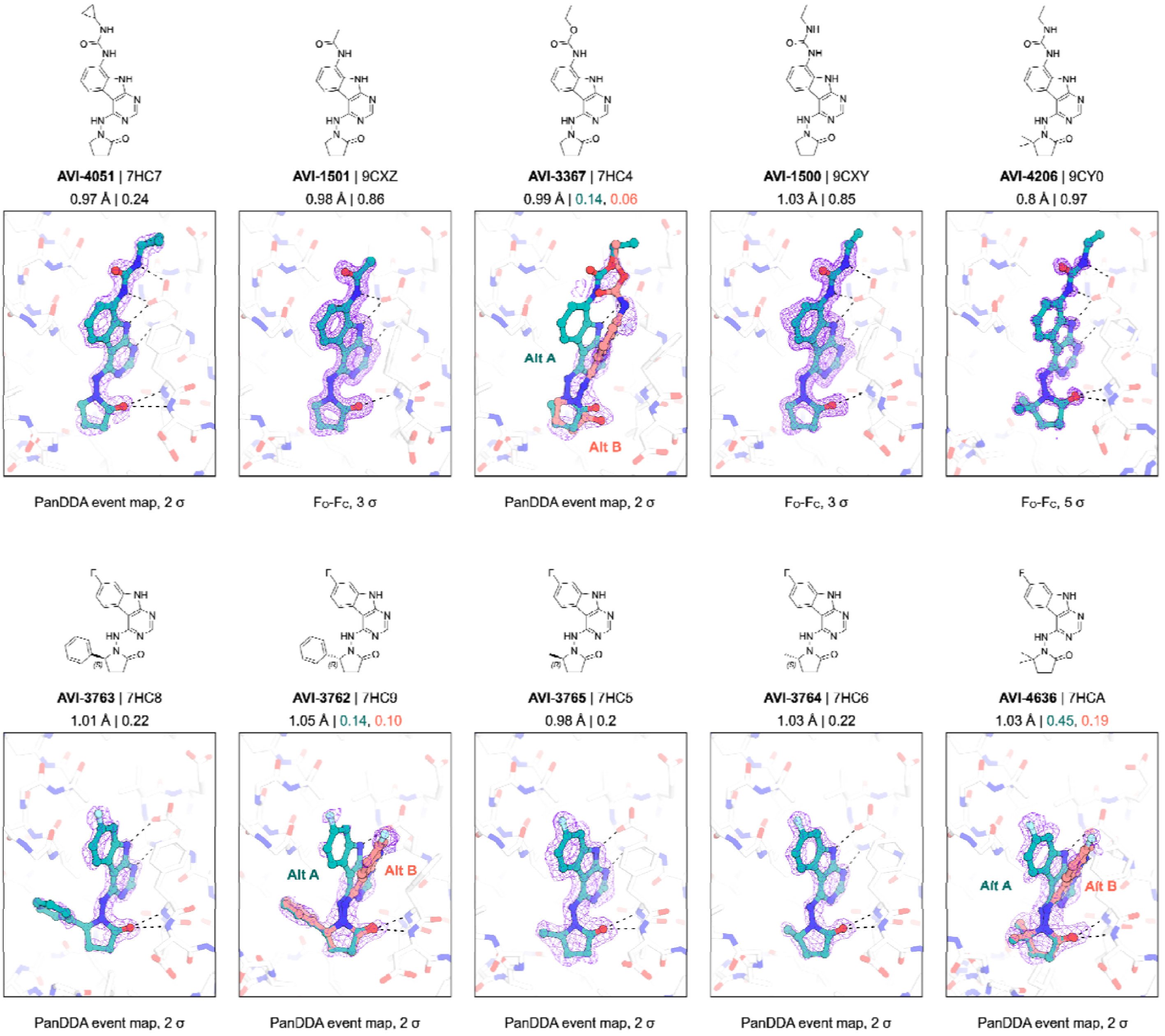

Figure 1 - Figure Supplement 1 - X-ray density for ligand modeling. Ligands were modeled using either traditional F_O-F_C electron density maps (AVI-1500, AVI-1501, AVI-4206) or PanDDA event maps (AVI-4051, AVI-3367, AVI-3763, AVI-3762, AVI-3765, AVI-3764 and AVI-4636). The diffraction resolution and refined occupancy are indicated for each ligand. The occupancy is indicated for each confirmation when multiple ligand poses were modeled.

59

bioRxiv preprint doi: https://doi.org/10.1101/2024.08.08.606661; this version posted October 6, 2025. The copyright holder for this preprint (which was not certified by peer review) is the author/funder. This article is a US Government work. It is not subject to copyright under 17 USC 105 and is also made available for use under a CC0 license.

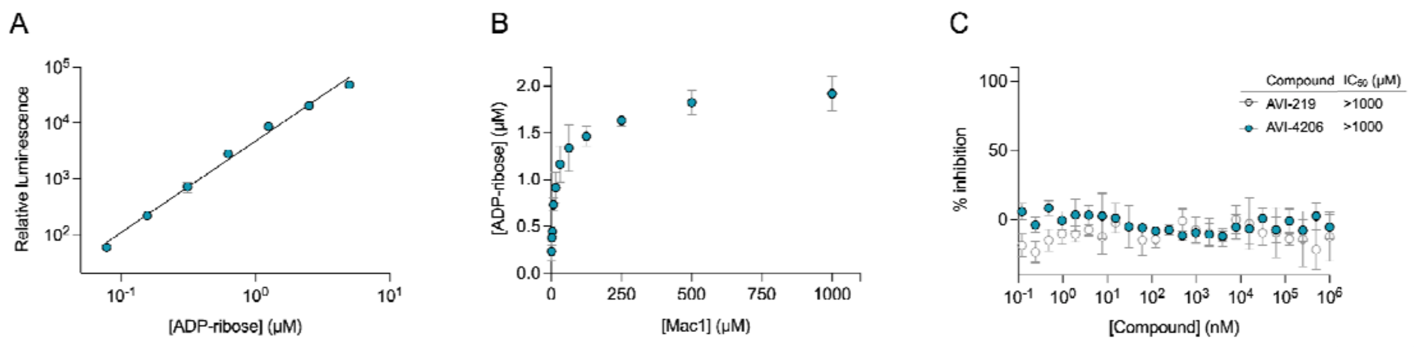

Figure 1 - Figure Supplement 2. AVI-4206 and AVI-219 inhibition of Mac1 determined using auto-mono-ADP-ribosylated PARP10 as a substrate.

(A) Standard curve of ADP-ribose detected using 100 nM NUDT5 and the AMP-Glo assay kit. Data are presented mean ± SD for four technical replicates. Data were fitted with a power function in the form $y = kx^a$ using non-linear regression (gray line).

(B) Titration of Mac1 with auto-mono-ADP-ribosylated PARP10. The concentration of PARP10 was 10 µM based on absorbance at 280 nm, but the titration indicated that the concentration of ADP-ribose released by Mac1 was five-fold lower (~2 µM). Data are presented mean ± SD for four technical replicates.

(C) Counterscreen of compounds against 100 nM NudT5 with 2 µM ADP-ribose as a substrate. No inhibition was detected up to 1 mM compound. Data are presented mean ± SD for four technical replicates.

bioRxiv preprint doi: https://doi.org/10.1101/2024.08.08.606661; this version posted October 6, 2025. The copyright holder for this preprint (which was not certified by peer review) is the author/funder. This article is a US Government work. It is not subject to copyright under 17 USC 105 and is also made available for use under a CC0 license.

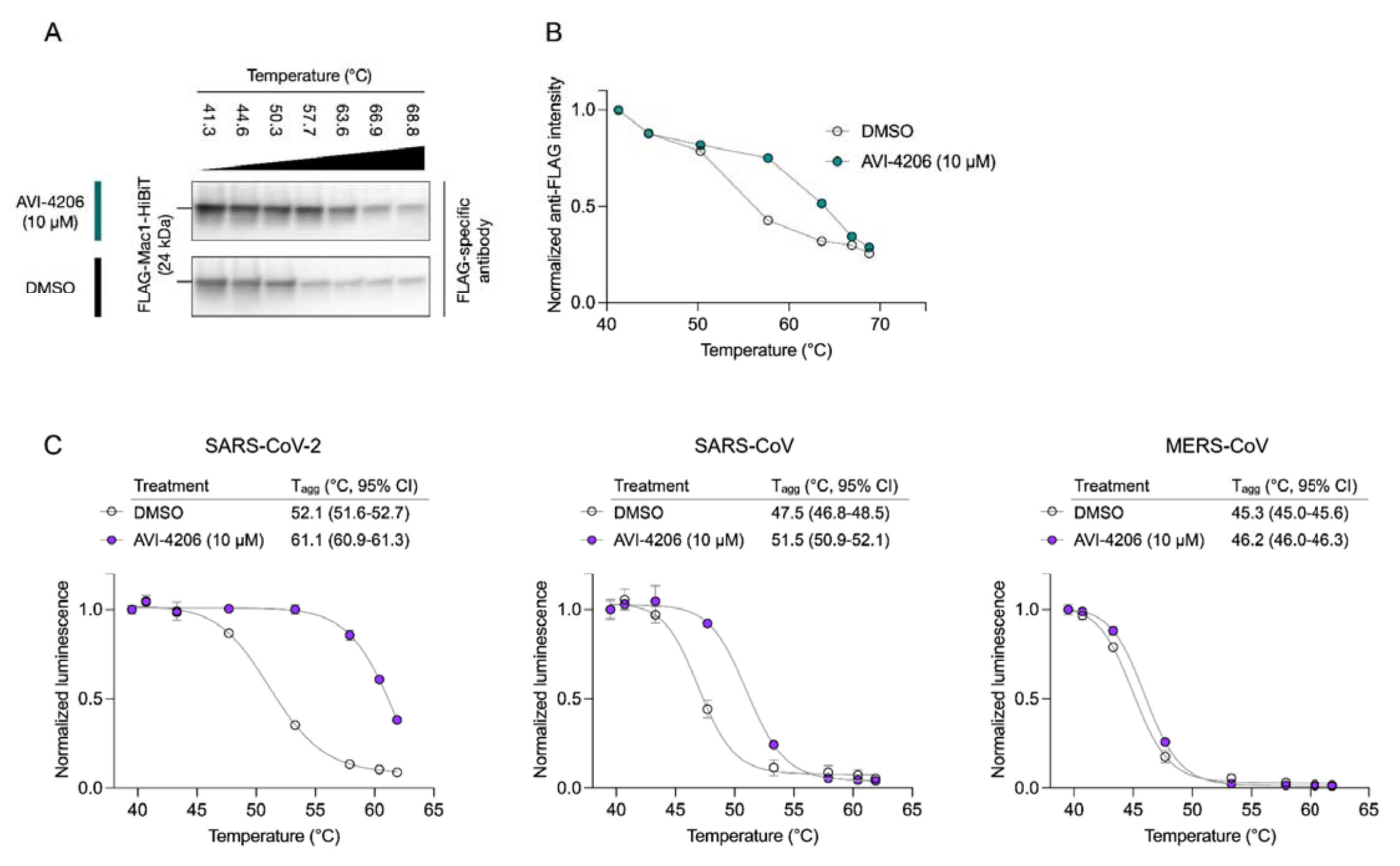

Figure 2 - Figure Supplement 1: AVI-4206 increases thermal stability of Mac1 in cells.
(A) CETSA-WB shows thermal stabilization of FLAG-tagged Mac1 protein after treatment of A549 cells with 10 μM of AVI-4206.
(B) Densitometry values were normalized to the lowest temperature for each treatment. Data are presented as a single densitometry measurement.
(C) *CETSA-nLuc shows differential stabilization of SARS-CoV2, SARS-CoV and MERS macrodomain proteins in A549 cells treated with 10 μM of AVI-4206. Data are presented as mean ± SD of two technical replicates. Data were fitted with a sigmoidal dose-response equation using non-linear regression (gray line) and the Tagg values are quoted with 95% confidence intervals*

bioRxiv preprint doi: https://doi.org/10.1101/2024.08.08.606661; this version posted October 6, 2025. The copyright holder for this preprint (which was not certified by peer review) is the author/funder. This article is a US Government work. It is not subject to copyright under 17 USC 105 and is also made available for use under a CC0 license.

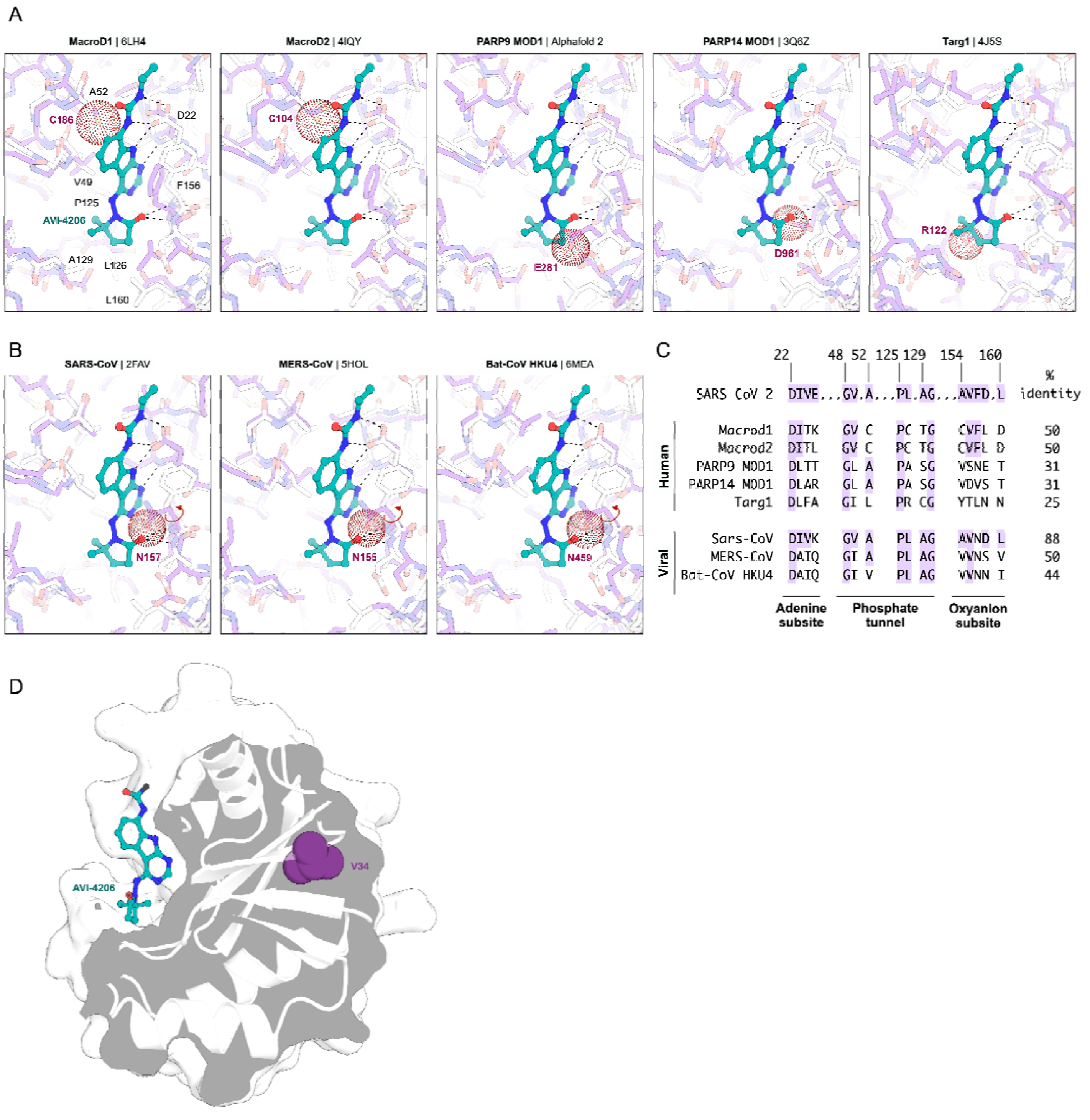

Figure 2 - Figure Supplement 2: **Alignment of AVI-4206 bound SARS-CoV-2 Mac1 with diverse Macrodomains suggests the origin of AVI-4206 selectivity**. (**A**) For human and (**B**) viral macrodomains, AVI-4206 is shown with teal spheres/sticks, SARS-CoV-2 Mac1 is shown with white sticks and the macrodomains being compared are shown with purple sticks. Selected Mac1 residues are labeled with black text, and the most important clashing human/viral residue is labeled and shown with red dots. For the clashing asparagine residue in the viral macrodomains (position equivalent to Phe156 in SARS-CoV-2 Mac1), rotation of the residue around the $\chi 1$ dihedral angle (shown with a red arrow) could relieve the clash and allow AVI-4206 binding. The human/viral macrodomain structures compared are bound to ADP-ribose, except for PARP9 MOD1, where no experimental structure is available and the Alphafold 2 model is shown. (**C**) The SARS-CoV-2 Mac1 sequence was aligned to human/viral macrodomains shown in (**A**) and (**B**) for SARS-CoV-2 Mac1 residues within 5 Å of AVI-4206. The SARS-CoV-2 residue numbering and the % identity for each sequence compared to SARS-CoV-2 Mac1 is indicated. (**D**) Surface view of Mac1 (PDB 9CY0) sliced through a plane containing AVI-4206 (teal sticks) and the site of the Val34Leu

bioRxiv preprint doi: https://doi.org/10.1101/2024.08.08.606661; this version posted October 6, 2025. The copyright holder for this preprint (which was not certified by peer review) is the author/funder. This article is a US Government work. It is not subject to copyright under 17 USC 105 and is also made available for use under a CC0 license.

mutation (purple spheres).

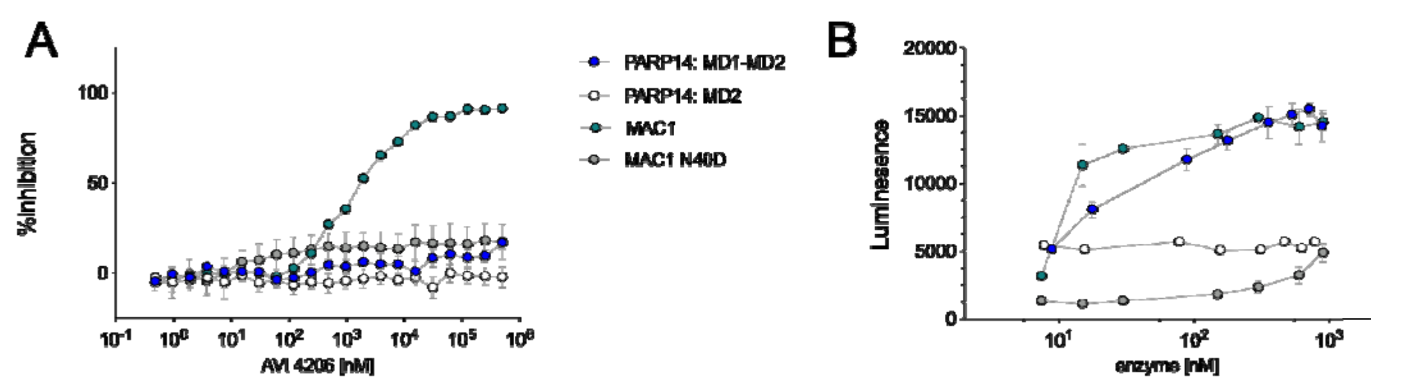

Figure 2 - Figure Supplement 3 PARP14 Macrodomain 1 activity is not inhibited by AVI-4206. (A) Titration of AVI-4206 shows full inhibition of WT Mac1, but no effect against catalytically active PARP14:MD1-MD2. Negative controls of Mac1 N40D and PARP14: MD2 are included. We note that the IC_{50} for WT Mac1 here is higher than in our other experiments, which we attribute to differences in the substrate preparation, the higher enzyme concentrations used (50 nM versus 10 nM), and the exact timing of the end point measurements. **(B)** Enzyme activity measurements to verify that WT Mac1 and PARP14:MD1-MD2 are active and that PARP14:MD2 and Mac1 N40D are inactive.

bioRxiv preprint doi: https://doi.org/10.1101/2024.08.08.606661; this version posted October 6, 2025. The copyright holder for this preprint (which was not certified by peer review) is the author/funder. This article is a US Government work. It is not subject to copyright under 17 USC 105 and is also made available for use under a CC0 license.

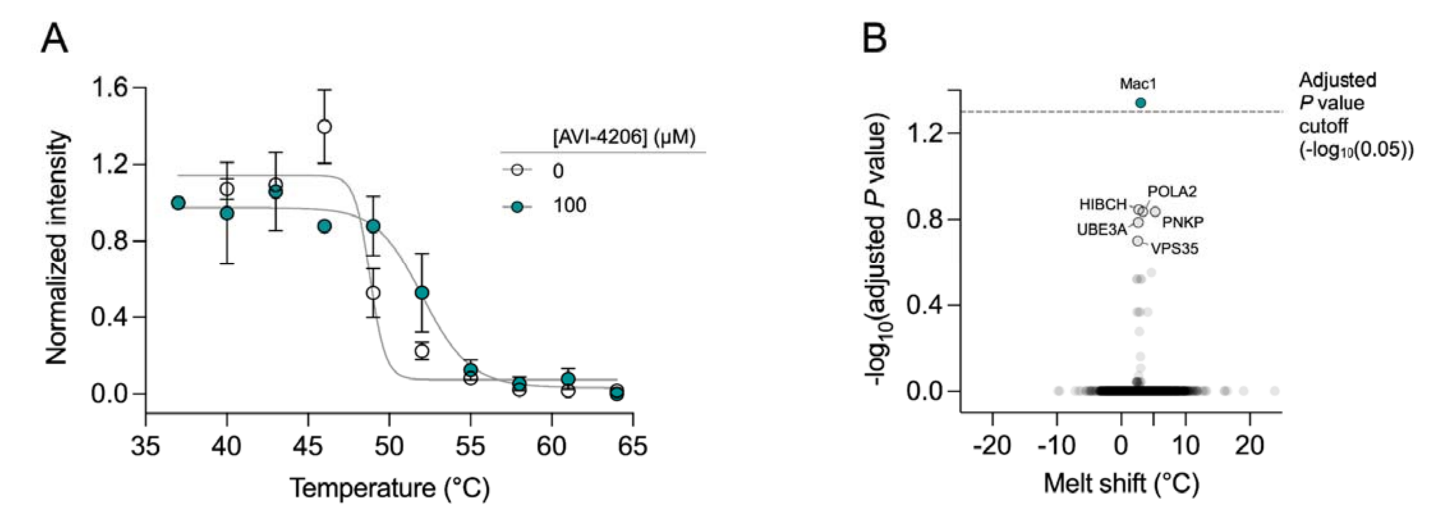

Figure 2 - Figure Supplement 4: **Thermal proteome profiling in A549 cellular lysates.**
(A) Melting curve for Mac1 in A549 lysates treated in duplicate with either DMSO or 100 µM of AVI-4206. Data were normalized to the mean intensity at 37°C. Data were fitted with a sigmoidal dose-response equation using non-linear regression (gray line).
(B) Volcano plot of the statistical significance and degree of melting temperature shift for all proteins with high quality melting curves (n= 3,446 proteins). Teal circles indicate proteins with a statistically significant shift in melting temperature (adjusted *P* value < 0.05). The highest non-significant proteins are labeled and do not have obvious functional overlap with macrodomains.

bioRxiv preprint doi: https://doi.org/10.1101/2024.08.08.606661; this version posted October 6, 2025. The copyright holder for this preprint (which was not certified by peer review) is the author/funder. This article is a US Government work. It is not subject to copyright under 17 USC 105 and is also made available for use under a CC0 license.

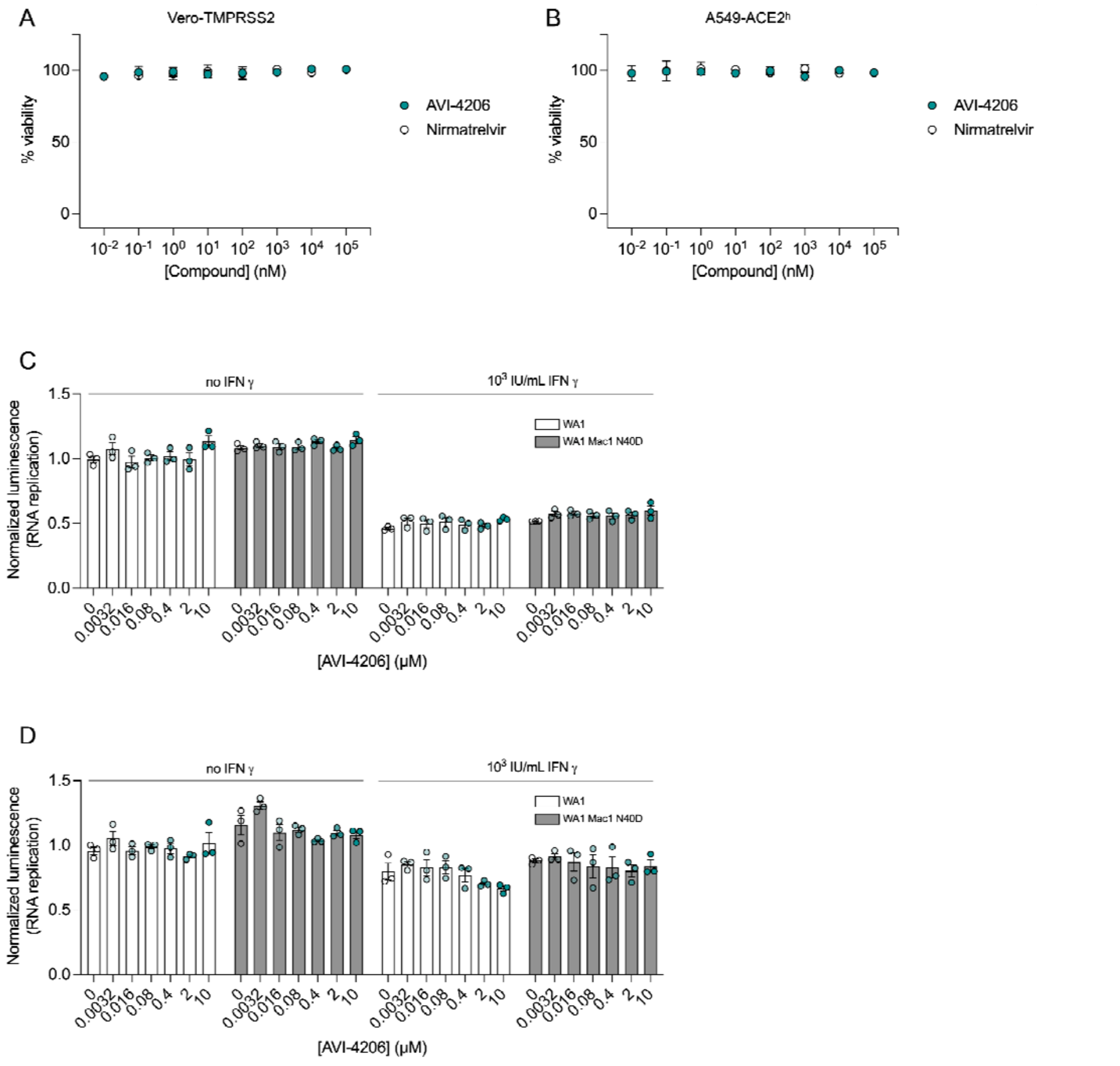

Figure 3 - Figure Supplement 1: AVI-4206 has limited antiviral efficacy and no cytotoxicity in cellular models of infection.

(A and B) Drug cytotoxicity of AVI-4206 in Vero-TMPRSS2 (A) and A549 ACE2^h (B) was measured using the CellTiter-Glo® viability assay. Graphs represent the mean ± SD of three biological replicates each conducted in triplicate.

(C and D) Luciferase readout of VAT (C) and A549 ACE2^h (D) cells infected with WA1 or WA1 Mac1 N40D replicons and treated with or without AVI-4206 and IFN-□ at indicated concentrations. Results are plotted as normalized mean ± SD luciferase values of a representative biological experiment containing three technical replicates.

bioRxiv preprint doi: https://doi.org/10.1101/2024.08.08.606661; this version posted October 6, 2025. The copyright holder for this preprint (which was not certified by peer review) is the author/funder. This article is a US Government work. It is not subject to copyright under 17 USC 105 and is also made available for use under a CC0 license.

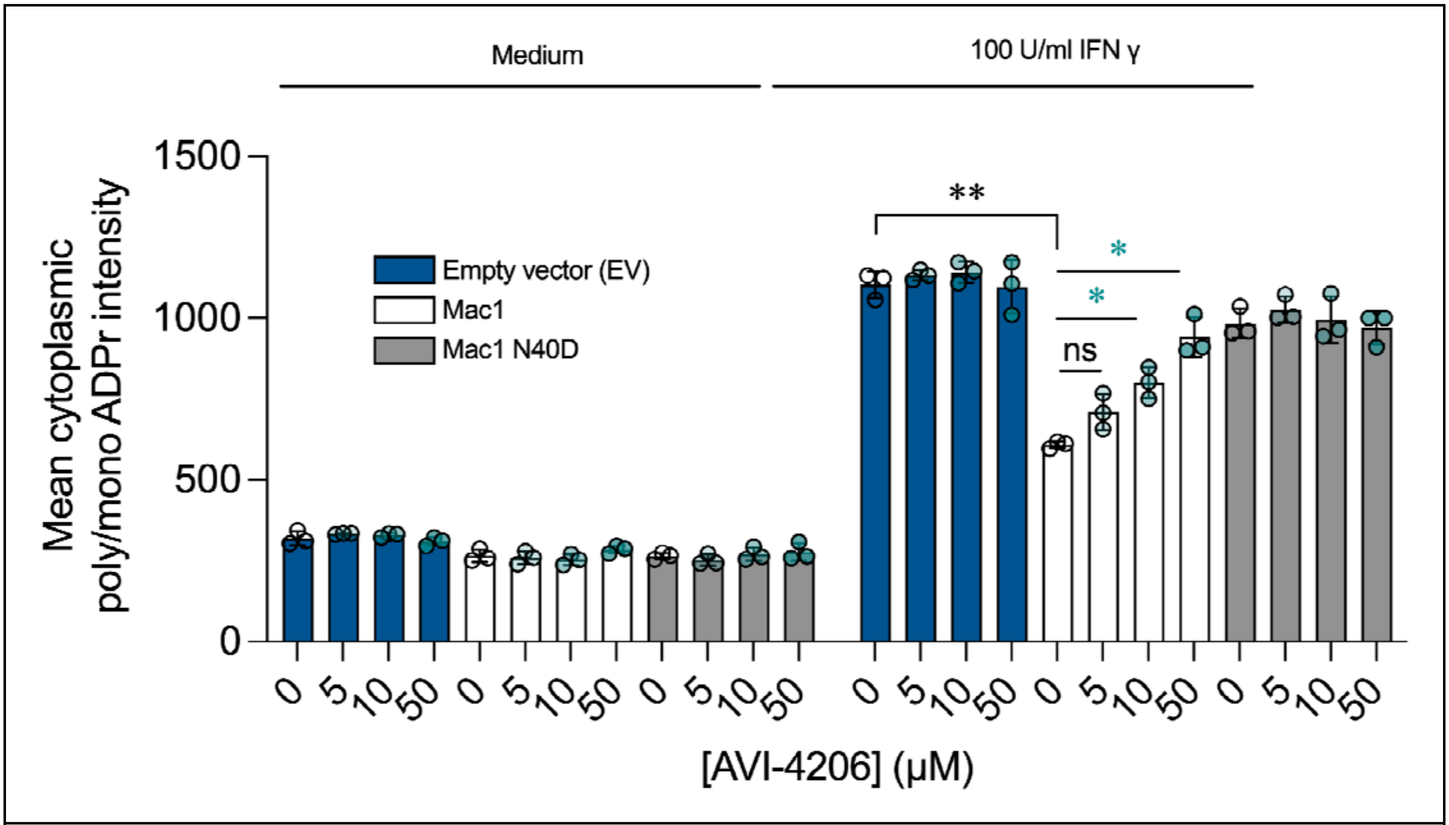

Figure 3 - Figure Supplement 2. **Relative mean cytoplasmic poly/mono ADPr intensity of A549 cells stably expressing Mac1 and Mac1-N40D treated with IFN-γ and/or AVI-4206**. DMSO-treated cells are shown as vehicle control. Poly/mono ADPr signal comes from Poly/Mono-ADP Ribose (E6F6A) Rabbit mAb (CST, 83732S) staining. Data shown as mean values ± SD; At least 10000 cells were analyzed each group, from triplicate wells. Two-tailed student's t-test were used to compare ADPr intensity levels of each treatment. *, $p < 0.05$; **, $p < 0.01$;

bioRxiv preprint doi: https://doi.org/10.1101/2024.08.08.606661; this version posted October 6, 2025. The copyright holder for this preprint (which was not certified by peer review) is the author/funder. This article is a US Government work. It is not subject to copyright under 17 USC 105 and is also made available for use under a CC0 license.

79

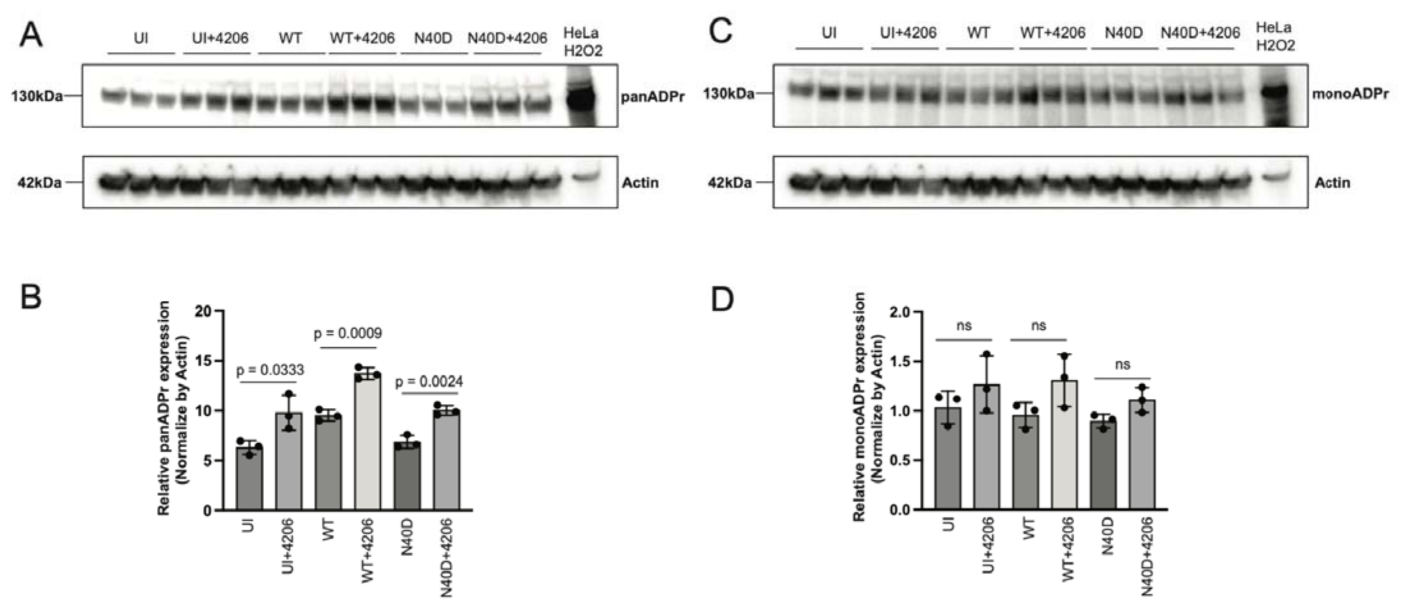

Figure 3 - Figure Supplement 3: **ADP-ribosylation profiling during infection by Western Blot.** (A) Immunoblot showing pan-ADP-ribose (panADPr) levels in Calu-3 cells under indicated infection conditions. UI = uninfected cells, WT = cells infected with WA1, N40D = cells infected with WA1 NSP3 Mac1 N40D, with or without 100 µM AVI-4206 treatment. HeLa cells treated with H_2O_2 were included as a positive control for the ADPr signal. Actin serves as a loading control. (B) Densitometric analysis of panADPr levels normalized to actin. Data are presented as mean ± SD (n = 3), with p-values indicated. (C) Immunoblot showing mono-ADP-ribose (monoADPr) levels under the same conditions as (A). (D) Densitometric analysis of monoADPr levels normalized to actin. No statistically significant differences (ns) were observed between conditions. Data are shown as mean ± SD (n = 3).

80

bioRxiv preprint doi: https://doi.org/10.1101/2024.08.08.606661; this version posted October 6, 2025. The copyright holder for this preprint (which was not certified by peer review) is the author/funder. This article is a US Government work. It is not subject to copyright under 17 USC 105 and is also made available for use under a CC0 license.

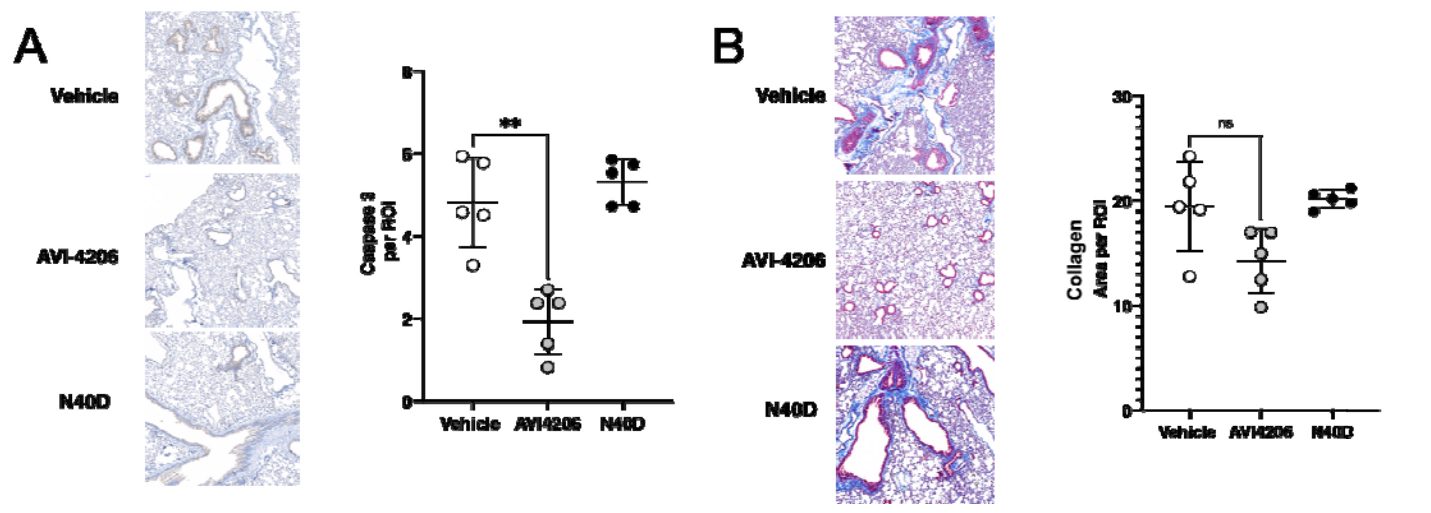

Figure 6 – Figure Supplement 1: **Lung Histology**: We examined determinants of tissue damage by either (A) caspase 3 staining for apoptosis or (B) Masson's Trichrome stain for collagen deposition and pulmonary fibrosis. In the lungs of AVI-4206 treated animals, apoptosis is significantly reduced compared to the lungs of the vehicle cohort. While collagen deposition in the lungs of AVI-4206 treated animals is trending lower, the result is not significant. There is no difference in pathology between the N40D cohort and vehicle-treated cohort with these markers. This could suggest that AVI-4206 provides an additional mechanism that results in protection.

bioRxiv preprint doi: https://doi.org/10.1101/2024.08.08.606661; this version posted October 6, 2025. The copyright holder for this preprint (which was not certified by peer review) is the author/funder. This article is a US Government work. It is not subject to copyright under 17 USC 105 and is also made available for use under a CC0 license.

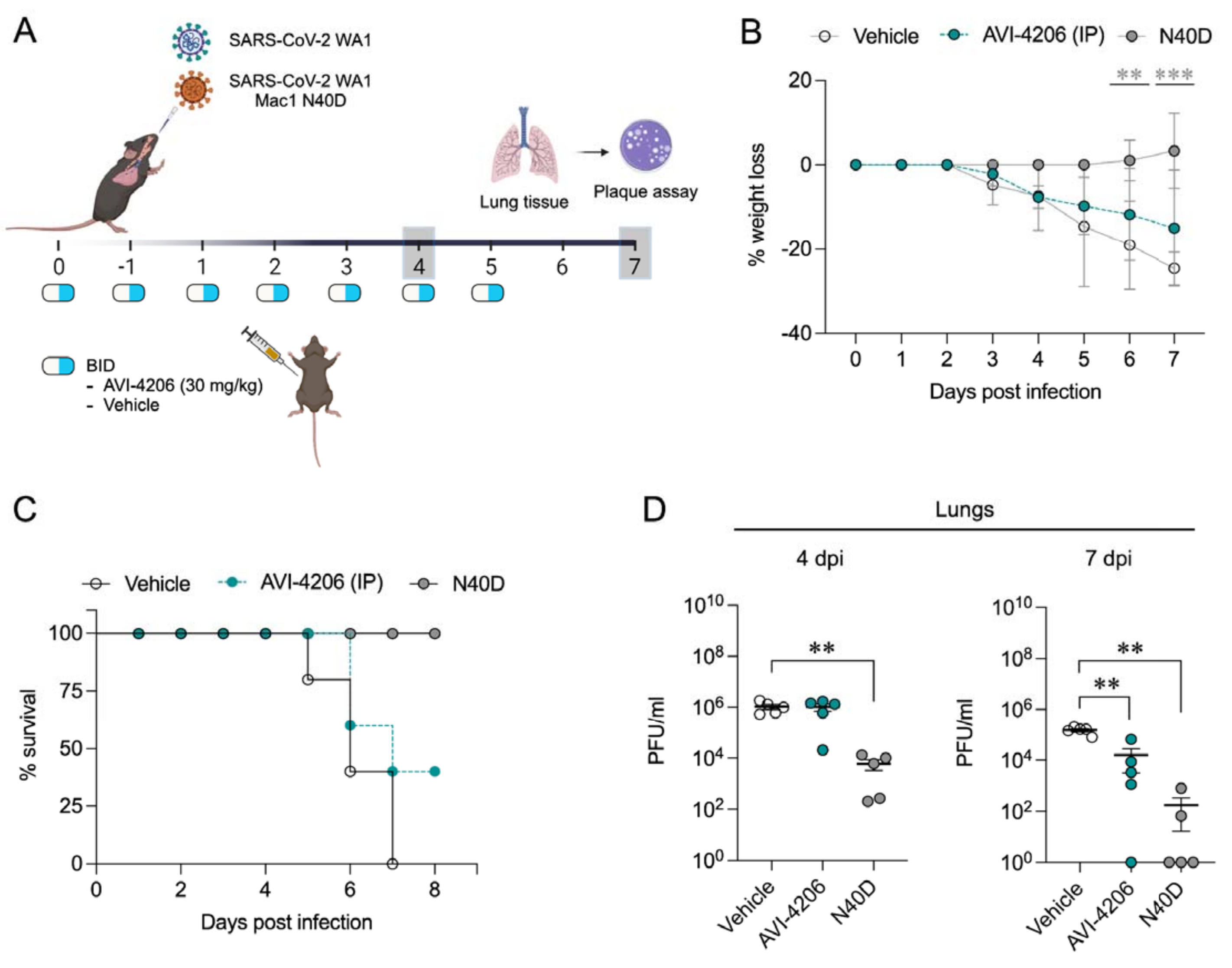

Figure 6 – Figure Supplement 2: **Lower dose AVI-4206 reduces viral replication and increases survival *in vivo*.**
(A) K18-hACE2 mice were intranasally infected SARS-CoV-2 WA1 or SARS-CoV-2 WA1 Mac1 N40D mutant. Mice were treated as indicated with AVI-4206 (BID, 30 mg/kg) or vehicle. Each group was composed of n=10 mice (5 mice per time point).
(B) The percent body weight loss is presented as mean ± SD. **, $P < 0.01$; ***, $P < 0.001$ by two-tailed Student's t-test relative to the vehicle control at each timepoint.

(C) Survival curve based on the percent body weight loss humane endpoint.
(D) Viral load in the lung at indicated time points is presented as mean ± s.e.m. **, $P < 0.01$ by Mann Whitney's test relative to the vehicle control.

bioRxiv preprint doi: https://doi.org/10.1101/2024.08.08.606661; this version posted October 6, 2025. The copyright holder for this preprint (which was not certified by peer review) is the author/funder. This article is a US Government work. It is not subject to copyright under 17 USC 105 and is also made available for use under a CC0 license.

36

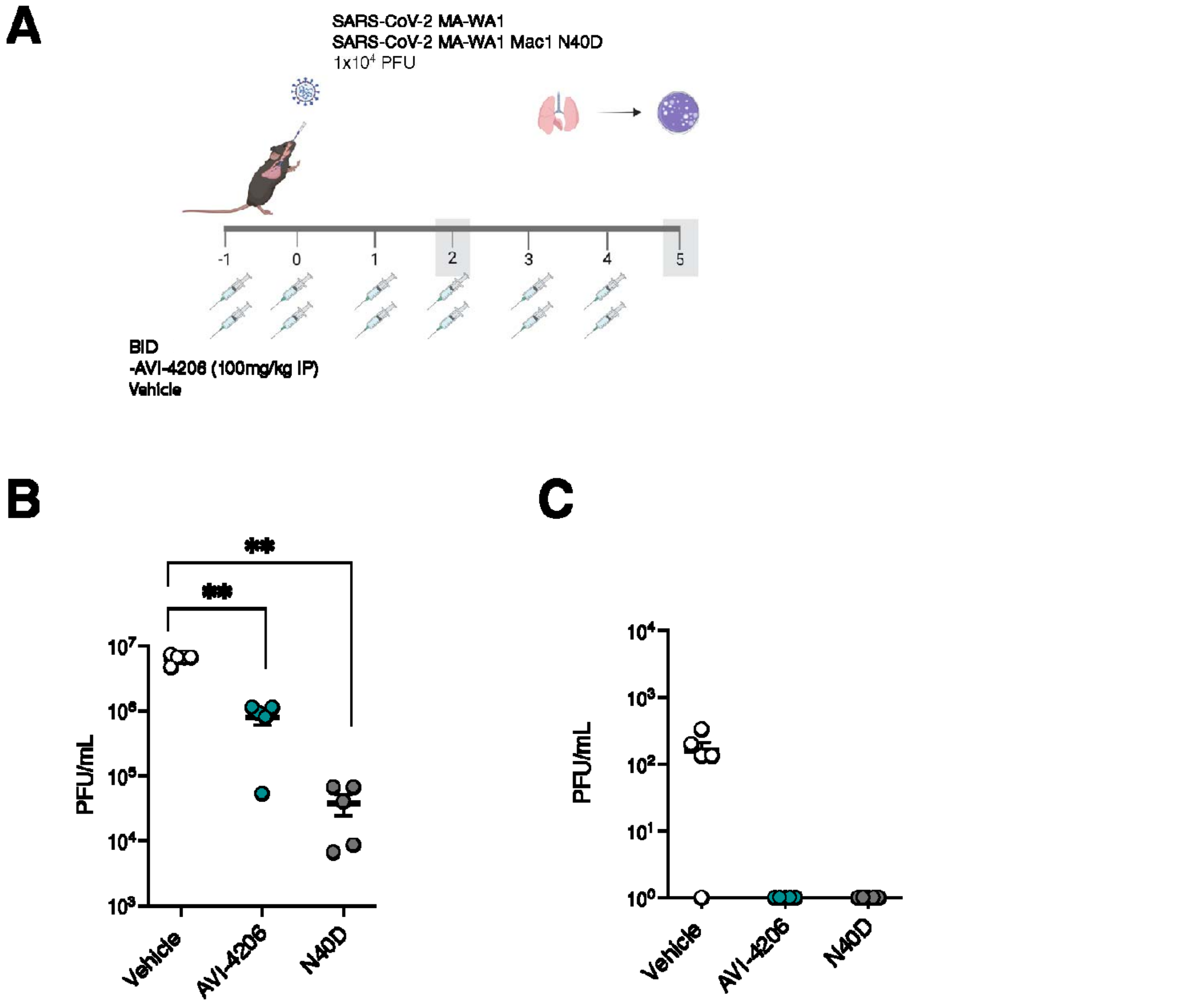

Figure 6 – Figure Supplement 3**: AVI-4206 suppresses replication of mouse-adapted SARS-CoV-2 in wild-type mice.**

(A) Wild-type mice were intranasally infected with SARS-CoV-2 and treated with AVI-4206, AVI-6451, or vehicle (n = 10 per group). Mice infected with the WA1 N40D mutant served as a positive control (n = 10). Lung tissues were collected at designated time points for viral titer analysis using a plaque assay.
(B) (C) Viral Load in the Lungs of Wild-Type Mice Treated with AVI-4206 (B, day2; C, day5).**, P < 0.01 by Mann Whitney's test relative to the vehicle control.

37
