# Supplementary material for: The Mac1 ADP-ribosylhydrolase is a Therapeutic Target for SARS-CoV-2
Source: bioRxiv. 2025 Jul 19:2024.08.08.606661. Originally published 2024 Aug 9. Preprint. [Version 3] doi: 10.1101/2024.08.08.606661 (PMC11326214; doi:10.1101/2024.08.08.606661)
Supplement: Supplementary file 1 [file NIHPP2024.08.08.606661v3-supplement-1.pdf]

bioRxiv preprint doi: https://doi.org/10.1101/2024.08.08.606661; this version posted July 19, 2025. The copyright holder for this preprint (which was not certified by peer review) is the author/funder, who has granted bioRxiv a license to display the preprint in perpetuity. It is made available under aCC-BY 4.0 International license.

# Supplementary information

**Supplementary Table1** - X-ray data collection and refinement deposition statistics.

| | **Ligand** | **AVI-1500** | **AVI-1501** | **AVI-3367** | **AVI-3765** | **AVI-3764** | **AVI-4051** | **AVI-3763** | **AVI-3762** | **AVI-4636** | **AVI-4206** |
|---|---|---|---|---|---|---|---|---|---|---|---|
| | PDB code | 9CXY | 9CXZ | 7HC4 | 7HC5 | 7HC6 | 7HC7 | 7HC8 | 7HC9 | 7HCA | 9CY0 |
| | Beam line | ALS 8.3.1 | ALS 8.3.1 | ALS 8.3.1 | ALS 8.3.1 | ALS 8.3.1 | ALS 8.3.1 | SSRL 9-2 | SSRL 9-2 | ALS 8.3.1 | ALS 8.3.1 |
| | Wavelength (Å) | 0.88557 | 0.88557 | 0.88557 | 0.88557 | 0.88557 | 0.88557 | 0.88557 | 0.88557 | 0.88557 | 0.77487 |
| | Resolution range (Å) | 39.9-1.03 (1.07-1.03) | 44.5-1.02 (1.06 - 1.02) | 39.7-1.00 (1.04 - 1.00) | 44.4-0.98 (1.02-0.98) | 44.5-1.03 (1.07-1.03) | 39.7-0.99 (1.03-0.99) | 39.7-1.01 (1.05-1.01) | 39.7-1.05 (1.09-1.05) | 44.47-1.03 (1.07-1.03) | 33.7-0.80 (0.83-0.80) |
| | Space group | $P4_3$ | $P4_3$ | $P4_3$ | $P4_3$ | $P4_3$ | $P4_3$ | $P4_3$ | $P4_3$ | $P4_3$ | P1 |
| | Unit cell (α β γ, a b c) | 88.8 88.8 39.5 90 90 90 | 88.9 88.9 39.4 90 90 90 | 88.8 88.8 39.5 90 90 90 | 88.8 88.8 39.5 90 90 90 | 88.8 88.8 39.6 90 90 90 | 88.8 88.8 39.4 90 90 90 | 88.8 88.8 39.6 90 90 90 | 88.8 88.8 39.6 9 0 90 90 | 88.9 88.9 39.3 90 90 90 | 30.3 39.8 64.4 89.9 77.8 90.1 |
| | Total reflections | 925861 (88714) | 1019499 (92950) | 1052667 (79477) | 1095419 (68284) | 981382 (94605) | 1075242 (76799) | 1049402 (101219) | 934650 (89428) | 996315 (94266) | 420519 (10537) |
| | Unique reflections | 151957 (14738) | 156086 (15234) | 165160 (15511) | 175278 (16099) | 152839 (14932) | 169798 (16087) | 161313 (15833) | 142525 (13787) | 151748 (14847) | 235488 (6157) |
| | Multiplicity | 6.1 (5.9) | 6.5 (6.1) | 6.4 (5.1) | 6.2 (4.1) | 6.4 (6.3) | 6.3 (4.7) | 6.5 (6.3) | 6.6 (6.4) | 6.6 (6.3) | 1.8 (1.7) |
| | Completeness (%) | 99.51 (96.98) | 99.59 (97.52) | 99.30 (93.65) | 99.13 (91.70) | 99.77 (98.28) | 99.44 (94.83) | 99.70 (98.49) | 98.59 (96.15) | 99.74 (98.30) | 75.89 (19.87) |
| | Mean I/sigma(I) | 13.96 (0.68) | 15.02 (1.04) | 13.32 (0.77) | 11.98 (0.58) | 9.03 (0.70) | 12.87 (0.79) | 12.16 (0.64) | 10.78 (0.66) | 13.86 (0.67) | 13.15 (1.22) |
| | Wilson B-factor | 13.38 | 12.91 | 12.4 | 12.14 | 11.92 | 12.15 | 11.98 | 12.53 | 14.12 | 6.69 |
| | R-merge | 0.0459 (2.25) | 0.0439 (1.39) | 0.0489 (1.38) | 0.0546 (1.61) | 0.0789 (1.73) | 0.0521 (1.34) | 0.0589 (2.38) | 0.0672 (2.3) | 0.0458 (2.24) | 0.0334 (0.463) |
| | R-meas | 0.0503 (2.47) | 0.0477 (1.52) | 0.0532 (1.54) | 0.0594 (1.85) | 0.0860 (1.88) | 0.0567 (1.5) | 0.0640 (2.60) | 0.0730 (2.51) | 0.0498 (2.44) | 0.0472 (0.655) |
| | R-pim | 0.0203 (0.995) | 0.0184 (0.607) | 0.0207 (0.665) | 0.0233 (0.879) | 0.0337 (0.751) | 0.0222 (0.669) | 0.0249 (1.03) | 0.0282 (0.979) | 0.0193 (0.961) | 0.0334 (0.463) |
| | $CC_{1/2}$ | 0.999 (0.463) | 0.999 (0.709) | 0.999 (0.547) | 0.999 (0.405) | 0.998 (0.537) | 0.999 (0.582) | 0.999 (0.466) | 0.999 (0.484) | 0.999 (0.424) | 0.997 (0.643) |
| | CC* | 1 (0.795) | 1 (0.911) | 1 (0.841) | 1 (0.759) | 1 (0.836) | 1 (0.858) | 1 (0.797) | 1 (0.808) | 1 (0.772) | 0.999 (0.885) |
| | Reflections used in refinement | 151730 (14738) | 156007 (15234) | 164947 (15512) | 174746 (16099) | 152688 (14932) | 169673 (16087) | 161090 (15833) | 142225 (13787) | 151557 (14847) | 235328 (6157) |
| | Reflections used for R-free | 7357 (789) | 7525 (740) | 7958 (711) | 8434 (781) | 7391 (787) | 8190 (751) | 7762 (741) | 6888 (690) | 7340 (790) | 11753 (303) |
| | R-work | 0.1436 (0.4057) | 0.1432 (0.3464) | 0.1416 (0.4193) | 0.1434 (0.4132) | 0.1439 (0.4514) | 0.1423 (0.3854) | 0.1502 (0.4026) | 0.1549 (0.5311) | 0.1552 (0.3774) | 0.1620 (0.2952) |
| | R-free | 0.1609 (0.4006) | 0.1594 (0.3372) | 0.1583 (0.4254) | 0.1587 (0.3953) | 0.1595 (0.4464) | 0.1580 (0.3611) | 0.1686 (0.3973) | 0.1773 (0.5333) | 0.1735 (0.3805) | 0.1884 (0.2959) |
| | CC(work) | 0.977 (0.759) | 0.975 (0.874) | 0.975 (0.807) | 0.975 (0.741) | 0.975 (0.823) | 0.974 (0.825) | 0.973 (0.785) | 0.974 (0.716) | 0.972 (0.737) | 0.954 (0.799) |
| | CC(free) | 0.971 (0.779) | 0.974 (0.881) | 0.967 (0.823) | 0.965 (0.777) | 0.966 (0.750) | 0.969 (0.847) | 0.963 (0.779) | 0.964 (0.677) | 0.964 (0.753) | 0.933 (0.763) |
| Number of atoms | Number of non-hydrogen atoms | 3082 | 3046 | 3759 | 3756 | 3658 | 3675 | 3488 | 3811 | 4148 | 6135 |
| | macromolecules | 2609 | 2615 | 3260 | 3253 | 3162 | 3177 | 2992 | 3315 | 3644 | 5240 |
| | ligands | 35 | 31 | 45 | 37 | 37 | 47 | 43 | 43 | 39 | 204 |
| | solvent | 446 | 406 | 472 | 480 | 473 | 470 | 469 | 469 | 481 | 783 |
| | Protein residues | 338 | 338 | 337 | 336 | 336 | 336 | 336 | 337 | 337 | 336 |
| RMS | Bonds (Å) | 0.175 | 0.144 | 0.038 | 0.006 | 0.006 | 0.006 | 0.006 | 0.11 | 0.056 | 0.317 |
| | Angles) (°) | 3.98 | 3.8 | 1.3 | 0.98 | 0.96 | 0.95 | 0.98 | 2.27 | 1.71 | 5.69 |
| Ramach-andran | Favored (%) | 99.1 | 98.8 | 99.4 | 99.4 | 99.4 | 99.4 | 99.4 | 99.4 | 99.1 | 98.8 |
| | Allowed (%) | 0.9 | 1.2 | 0.6 | 0.6 | 0.6 | 0.6 | 0.6 | 0.6 | 0.9 | 1.2 |
| | Outliers (%) | 0 | 0 | 0 | 0 | 0 | 0 | 0 | 0 | 0 | 0 |
| | Rotamer outliers (%) | 0 | 0 | 2.54 | 1.12 | 0.87 | 0.86 | 1.81 | 1.39 | 0.76 | 0.69 |
| | Clashscore | 0.76 | 1.51 | 2.13 | 2.28 | 2.03 | 2.65 | 2.15 | 4.49 | 2.85 | 3.11 |
| B-factor ($Å^2$) | Average | 21.5 | 20.92 | 19.02 | 18.79 | 18.04 | 19.08 | 19.44 | 19.55 | 22.95 | 9.88 |
| | Macromolecules | 19.43 | 19.14 | 17.11 | 16.99 | 16.18 | 17.19 | 17.55 | 17.73 | 20.99 | 8.95 |
| | Ligands | 18.01 | 16.17 | 17.48 | 12.72 | 13.56 | 17.7 | 17.35 | 18.26 | 17.1 | 6.82 |
| | Solvent | 33.82 | 32.71 | 32.34 | 31.27 | 30.7 | 31.88 | 31.62 | 32.52 | 38.09 | 16.58 |

bioRxiv preprint doi: https://doi.org/10.1101/2024.08.08.606661; this version posted July 19, 2025. The copyright holder for this preprint (which was not certified by peer review) is the author/funder, who has granted bioRxiv a license to display the preprint in perpetuity. It is made available under aCC-BY 4.0 International license.

**Supplementary Table2 -** Eurofins scanEDGE kinase assay shows no inhibition greater than >35% at 10 μM across a panel of diverse kinases

| Compound Name | Entrez Gene Symbol | Percent Control |
|---|---|---|
| AVI-4206 | ABL1 | 74 |
| AVI-4206 | ABL1 | 95 |
| AVI-4206 | ABL1 | 67 |
| AVI-4206 | ABL1 | 78 |
| AVI-4206 | ACVR1B | 91 |
| AVI-4206 | CABC1 | 100 |
| AVI-4206 | AKT1 | 80 |
| AVI-4206 | AKT2 | 100 |
| AVI-4206 | ALK | 69 |
| AVI-4206 | AURKA | 95 |
| AVI-4206 | AURKB | 77 |
| AVI-4206 | AXL | 84 |
| AVI-4206 | BMPR2 | 92 |
| AVI-4206 | BRAF | 70 |
| AVI-4206 | BRAF | 99 |
| AVI-4206 | BTK | 100 |
| AVI-4206 | CDK19 | 99 |
| AVI-4206 | CDK2 | 86 |
| AVI-4206 | CDK3 | 100 |
| AVI-4206 | CDK7 | 99 |
| AVI-4206 | CDK9 | 100 |

bioRxiv preprint doi: https://doi.org/10.1101/2024.08.08.606661; this version posted July 19, 2025. The copyright holder for this preprint (which was not certified by peer review) is the author/funder, who has granted bioRxiv a license to display the preprint in perpetuity. It is made available under aCC-BY 4.0 International license.

| | | |
|---|---|---|
| AVI-4206 | CHEK1 | 90 |
| AVI-4206 | CSF1R | 84 |
| AVI-4206 | CSNK1D | 88 |
| AVI-4206 | CSNK1G2 | 97 |
| AVI-4206 | DCLK1 | 91 |
| AVI-4206 | DYRK1B | 99 |
| AVI-4206 | EGFR | 91 |
| AVI-4206 | EGFR | 95 |
| AVI-4206 | EPHA2 | 100 |
| AVI-4206 | ERBB2 | 91 |
| AVI-4206 | ERBB4 | 100 |
| AVI-4206 | MAPK3 | 98 |
| AVI-4206 | PTK2 | 89 |
| AVI-4206 | FGFR2 | 100 |
| AVI-4206 | FGFR3 | 97 |
| AVI-4206 | FLT3 | 97 |
| AVI-4206 | GSK3B | 81 |
| AVI-4206 | IGF1R | 100 |
| AVI-4206 | CHUK | 82 |
| AVI-4206 | IKBKB | 93 |
| AVI-4206 | INSR | 68 |
| AVI-4206 | JAK2 | 94 |
| AVI-4206 | JAK3 | 93 |
| AVI-4206 | MAPK8 | 88 |

bioRxiv preprint doi: https://doi.org/10.1101/2024.08.08.606661; this version posted July 19, 2025. The copyright holder for this preprint (which was not certified by peer review) is the author/funder, who has granted bioRxiv a license to display the preprint in perpetuity. It is made available under aCC-BY 4.0 International license.

| | | |
|---|---|---|
| AVI-4206 | MAPK9 | 73 |
| AVI-4206 | MAPK10 | 88 |
| AVI-4206 | KIT | 100 |
| AVI-4206 | KIT | 100 |
| AVI-4206 | KIT | 89 |
| AVI-4206 | STK11 | 39 |
| AVI-4206 | MAP3K4 | 96 |
| AVI-4206 | MAPKAPK2 | 85 |
| AVI-4206 | MARK3 | 94 |
| AVI-4206 | MAP2K1 | 55 |
| AVI-4206 | MAP2K2 | 60 |
| AVI-4206 | MET | 98 |
| AVI-4206 | MKNK1 | 68 |
| AVI-4206 | MKNK2 | 70 |
| AVI-4206 | MAP3K9 | 100 |
| AVI-4206 | MAPK14 | 99 |
| AVI-4206 | MAPK11 | 91 |
| AVI-4206 | PAK1 | 100 |
| AVI-4206 | PAK2 | 88 |
| AVI-4206 | PAK4 | 100 |
| AVI-4206 | CDK16 | 83 |
| AVI-4206 | PDGFRA | 41 |
| AVI-4206 | PDGFRB | 91 |
| AVI-4206 | PDPK1 | 85 |

bioRxiv preprint doi: https://doi.org/10.1101/2024.08.08.606661; this version posted July 19, 2025. The copyright holder for this preprint (which was not certified by peer review) is the author/funder, who has granted bioRxiv a license to display the preprint in perpetuity. It is made available under aCC-BY 4.0 International license.

| | | |
|---|---|---|
| AVI-4206 | PIK3C2B | 100 |
| AVI-4206 | PIK3CA | 100 |
| AVI-4206 | PIK3CG | 100 |
| AVI-4206 | PIM1 | 100 |
| AVI-4206 | PIM2 | 86 |
| AVI-4206 | PIM3 | 100 |
| AVI-4206 | PRKACA | 84 |
| AVI-4206 | PLK1 | 86 |
| AVI-4206 | PLK3 | 90 |
| AVI-4206 | PLK4 | 91 |
| AVI-4206 | PRKCE | 76 |
| AVI-4206 | RAF1 | 95 |
| AVI-4206 | RET | 100 |
| AVI-4206 | RIOK2 | 61 |
| AVI-4206 | ROCK2 | 74 |
| AVI-4206 | RPS6KA3 | 67 |
| AVI-4206 | NUAK2 | 68 |
| AVI-4206 | SRC | 82 |
| AVI-4206 | SRPK3 | 92 |
| AVI-4206 | TGFBR1 | 84 |
| AVI-4206 | TEK | 100 |
| AVI-4206 | NTRK1 | 99 |
| AVI-4206 | TSSK1B | 100 |
| AVI-4206 | TYK2 | 100 |

bioRxiv preprint doi: https://doi.org/10.1101/2024.08.08.606661; this version posted July 19, 2025. The copyright holder for this preprint (which was not certified by peer review) is the author/funder, who has granted bioRxiv a license to display the preprint in perpetuity. It is made available under aCC-BY 4.0 International license.

| | | |
|---|---|---|
| AVI-4206 | ULK2 | 100 |
| AVI-4206 | KDR | 45 |
| AVI-4206 | STK32C | 85 |
| AVI-4206 | ZAP70 | 91 |

**Supplementary Table3** Pharmacokinetic parameters for AVI-4206 following IV (10 mg/kg), PO (50 mg/kg), and IP (100 mg/kg) doses in male CD1 mice (n = 3 per group).

| IV (10 mg/kg) | | | PO (50 mg/kg) | | | IP (100 mg/kg) | | |
|---|---|---|---|---|---|---|---|---|
| parameter | units | value | parameter | units | value | parameter | units | value |
| CL | ml/min/kg | 69.5 | $T_{max}$ | hr | 1.0 | $T_{max}$ | hr | 0.250 |
| $V_{ss}$ | l/kg | 1.32 | $C_{max}$ | ng/ml | 94.0 | $C_{max}$ | ng/ml | 38067 |
| $T_{1/2}$ | hr | 0.833 | $T_{1/2}$ | hr | 4.69 | $T_{1/2}$ | hr | 3.17 |
| $AUC_{last}$ | hr*ng/ml | 2396 | $AUC_{last}$ | hr*ng/ml | 433 | $AUC_{last}$ | hr*ng/ml | 40998 |
| $AUC_{INF}$ | hr*ng/ml | 2399 | $AUC_{INF}$ | hr*ng/ml | 442 | $AUC_{INF}$ | hr*ng/ml | 41003 |
| $MRT_{INF}$ | hr | 0.316 | F | % | 3.69 | F | % | 171 |

Liver blood flow (mouse) = 120 ml/min/kg.

**Supplementary Table4** - ADMET panel shows no antagonist response greater than >15% at 10 μM.

bioRxiv preprint doi: https://doi.org/10.1101/2024.08.08.606661; this version posted July 19, 2025. The copyright holder for this preprint (which was not certified by peer review) is the author/funder, who has granted bioRxiv a license to display the preprint in perpetuity. It is made available under aCC-BY 4.0 International license.

| Assay | Ligand or Substrate | Antagonist Response at 10 µM |
|---|---|---|
| Cholinesterase, Acetyl, ACES | Acetylthiocholine | 4.1 |
| Peptidase Bleomycin Hydrolase (BLMH) | Met-AMC | -0.4 |
| Peptidase, CAN1 (CANPL1, Calpain-1) | Casein-FITC | 5 |
| Peptidase, CTSB (Cathepsin B) | Boc-Leu-Arg-Arg-AMC | -17.5 |
| Peptidase, CTSG (Cathepsin G) | Suc-Ala-Ala-Pro-Phe-AMC | -3.2 |
| Peptidase, CTSH (Cathepsin H) | L-Arg-AMC | -13.7 |
| Peptidase, CTSK (Cathepsin K) | Z-Phe-Arg-AMC | -5 |
| Peptidase, CTSL (Cathepsin L) | Z-Phe-Arg-AMC | 2.6 |
| Peptidase, CTSS (Cathepsin S) | Z-Leu-Arg-AMC | -50.7 |
| Peptidase, CTSL2 (Cathepsin L2) | Z-Leu-Arg-AMC | -1.2 |
| Peptidase, CTSZ (Cathepsin Z) | Mca-Arg-Pro-Pro-Gly-Phe-Ser-Ala-Phe-Lys(Dnp)-OH | -7.1 |
| Peptidase, Chymase | Suc-Ala-Ala-Pro-Phe-AMC | -5.1 |
| Peptidase, Chymotrypsin | Suc-Ala-Ala-Pro-Phe-AMC | -0.7 |
| Peptidase, Factor VIIa | N-CH3-SO2-D-Phe-Gly-Arg-pNA | -3.3 |
| Peptidase Kallikrein Plasma | Z-FR-AMC | 7.9 |
| Peptidase, PLAU (Urokinase) | Z-Gly-Gly-Arg-AMC | 11.2 |
| Cyclooxygenase COX-1 | Arachidonic acid | 0.6 |
| Cyclooxygenase COX-2 | Arachidonic Acid | 5.7 |
| Monoamine Oxidase MAO-A | Kynuramine | 3.3 |
| Phosphodiesterase PDE3A | FAM-cAMP | 0.6 |
| Phosphodiesterase PDE4D2 | FAM-cAMP | 0.8 |
| Peptidase, CASP2 (Caspase 2) | Z-VDVAD-AFC | 4.1 |

bioRxiv preprint doi: https://doi.org/10.1101/2024.08.08.606661; this version posted July 19, 2025. The copyright holder for this preprint (which was not certified by peer review) is the author/funder, who has granted bioRxiv a license to display the preprint in perpetuity. It is made available under aCC-BY 4.0 International license.

| | | |
|---|---|---|
| Peptidase, CASP4 (Caspase 4) | Ac-LEVD-AFC | -2.9 |
| Peptidase, CASP5 (Caspase 5) | Ac-WEHD-AFC | -6.6 |
| Peptidase, Prolyl Oligopeptidase (POP) | Z-Gly-Pro-AMC | -0.5 |
| Peptidase, Plasmin | H-D-Val-Leu-Lys-pNA | -1.5 |
| Peptidase, Thrombin | Z-Gly-Pro-Arg-AMC | 0.4 |
| Peptidase, Tissue Plasminogen Activator (tPA) | N-alpha-Z-D-Arg-Gly-Arg-pNA | 1.5 |
| Peptidase, Trypsin | Z-Gly-Pro-Arg-AMC | -1.6 |
| Peptidase, Tryptase | Z-Gly-Pro-Arg-AMC | -0.5 |
| Peptidase, ELA2 (Neutrophil Elastase 2) | N-MeOSuc-Ala-Ala-Pro-Val-pNA | -3 |
| Peptidase, ELA1 (Pancreatic Elastase 1) | N-MeOSuc-Ala-Ala-Pro-Val-pNA | -2.2 |
| Protein Tyrosine Kinase, LCK | Poly(Glu:Tyr) | -1.4 |
| Peptidase, Dipeptidyl Peptidase 4 (DPP4, DPP IV) | GP-AMC | 1.3 |
| Peptidase, CASP1 (Caspase 1) | Z-YUAD-AFC | -0.9 |
| Adenosine A2A | [3H]CGS-21680 | -1 |
| Adrenergic alpha1A | [3H]Prazosin | 2.1 |
| Adrenergic alpha2A | [3H]Rauwolscine | 4.9 |
| Adrenergic beta1 | [125I]Cyanopindolol | 2.4 |
| Adrenergic beta2 | [3H]CGP-12177 | -10.5 |
| Transporter, Norepinephrine (NET) | [125I]RTI-55 | -2.4 |
| Androgen (Testosterone) | [3H]Methyltrienolone | -1.5 |
| Calcium Channel L-Type, Dihydropyridine | [3H]Nitrendipine | -6.2 |
| Cannabinoid CB1 | [3H]SR141716A | 4.2 |
| Cannabinoid CB2 | [3H]WIN-55,212-2 | 6.7 |
| Cholecystokinin CCK1 (CCKA) | [125I]CCK-8 | -6.9 |

bioRxiv preprint doi: https://doi.org/10.1101/2024.08.08.606661; this version posted July 19, 2025. The copyright holder for this preprint (which was not certified by peer review) is the author/funder, who has granted bioRxiv a license to display the preprint in perpetuity. It is made available under aCC-BY 4.0 International license.

| | | |
|---|---|---|
| Dopamine D1 | [3H]SCH-23390 | -12.1 |
| Dopamine D2S | [3H]Spiperone | -15.8 |
| Transporter, Dopamine (DAT) | [125I]RTI-55 | 6 |
| Endothelin ETA | [125I]Endothelin-1 | 3 |
| GABAA, Flunitrazepam, Central | [3H]Flunitrazepam | -9.9 |
| Glucocorticoid | [3H]Dexamethasone | 6.9 |
| Glutamate, NMDA, Agonism | [3H]CGP-39653 | -2.9 |
| Histamine H1 | [3H]Pyrilamine | -12.8 |
| Histamine H2 | [125I]Aminopotentidine | 2.5 |
| Muscarinic M1 | [3H]N-Methylscopolamine | -8.1 |
| Muscarinic M2 | [3H]N-Methylscopolamine | -0.4 |
| Muscarinic M3 | [3H]N-Methylscopolamine | -2.6 |
| Opiate delta1 (OP1, DOP) | [3H]Naltrindole | -3.2 |
| Opiate kappa (OP2, KOP) | [3H]Diprenorphine | 6.3 |
| Opiate mu (OP3, MOP) | [3H]Diprenorphine | 8.2 |
| Potassium Channel [KA] | [125I]alpha-Dendrotoxin | 5.6 |
| Potassium Channel hERG, [3H]Dofetilide | [3H]Dofetilide | -4.7 |
| Serotonin (5-Hydroxytryptamine) 5-HT1A | [3H]8-OH-DPAT | -2.3 |
| Serotonin (5-Hydroxytryptamine) 5-HT1B | [3H]GR125743 | 2.2 |
| Serotonin (5-Hydroxytryptamine) 5-HT2A | [3H]Ketanserin | 8.3 |
| Serotonin (5-Hydroxytryptamine) 5-HT2B | [3H]Lysergic acid diethylamide (LSD) | -4.5 |
| Serotonin (5-Hydroxytryptamine) 5-HT3 | [3H]GR-65630 | -3.2 |
| Transporter, Serotonin (5-Hydroxytryptamine) (SERT) | [3H]Paroxetine | -2.2 |
| Sodium Channel Nav1.5 | [3H]BNZA | 0.3 |

bioRxiv preprint doi: https://doi.org/10.1101/2024.08.08.606661; this version posted July 19, 2025. The copyright holder for this preprint (which was not certified by peer review) is the author/funder, who has granted bioRxiv a license to display the preprint in perpetuity. It is made available under aCC-BY 4.0 International license.

| | | |
|---|---|---|
| Vasopressin V1A | [125I]PhenylacetylTyr(Me)PheGlnAsnArgProArgTyr | 2 |
| Nicotinic Acetylcholine alpha4beta2, Cytisine | [3H]Cytisine | 4.7 |

**Supplementary Table5** Macrodomain protein sequences

| Protein | Use | Sequence |
|---|---|---|
| hMacroD2 | HTRF | M**HHHHHH**SSGVDLGTENLYFQSYPSNKKKKVWREEKERLLKMTLEERRKEYLRDYIPLNSILSWKEEMKGKGQNDEENTQETSQVKKSLTEKVSLYRGDITLLEVDAIVNAANASLLGGGGVDGCIHRAAGPCLLAECRNLNGCDTGHAKITCGYDLPAKYVIHTVGPIARGHINGSHKEDLANCYKSSLKLVKENNIRSVAFPCISTGIYGFPNEPAAVIALNTIKEWLAKNHHEVDRIIFCVFLEVDFKIYKKKMNEFFSVDDNNEEEEDVEMKEDSDENGPEEKQSVEEMEEQSQDADGVNTVTVPGPASEEAVEDCKDEDFAKDENITKGGEVTDHSVRDQDHPDGQENDSTKNEIKIETESQSSYMETEELSSNQEDAVIVEQPEVIPLTEDQEEKEGEKAPGEDTPRMPGKSEGSSDLENTPGPDAGAQDEAKEQRNGTKGLNDIFEAQKIEWHE |
| Targ1 | HTRF | M**HHHHHH**SSGVDLGTENLYFQSMASSLNEDPEGSRITYVKGDLFACPKTDSLAHCISEDCRMGAGIAVLFKKKFGGVQELLNQQKKSGEVAVLKRDGRYIYYLITKKRASHKPTYENLQKSLEAMKSHCLKNGVTDLSMPRIGCGLDRLQWENVSAMIEEVFEATDIKITVYTL |
| Mac1 | HTRF | MSYY**HHHHHH**LESTSLYKKAGFLEVLFQGPEVNSFSGYLKLTDNVYIKNADIVEEAKKVKPTVVVNAANVYLKHGGGVAGALNKATNNAMQVESDDYIATNGPLKVGGSCVLSGHNLAKHCLHVVGPNVNKGEDIQLLKSAYENFNQHEVLLAPLLSAGIFGADPIHSLRVCVDTVRTNVYLAVFDKNLYDKLVSSFLEMKSEK |
| Mac1 | CETSA | MDYKDHDGDYKDHDIDYKDDDDKGGGSGGGSGGGSTIEVNSFSGYLKLTDNVYIKNADIVEEAKKVKPTVVVNAANVYLKHGGGVAGALNKATNNAMQVESDDYIATNGPLKVGGSCVLSGHNLAKHCLHVVGPNVNKGEDIQLLKSAYENFNQHEVLLAPLLSAGIFGADPIHSLRVCVDTVRTNVYLAVFDKNLYDKLVSSFLEMKGSVSGWRLFKKISGSEGRGSLLTCGDVEENPGPMVSELIKENMHMKLYMEGTVNNHHFKCTSEGEGKPYEGTQTMRIKAVEGGPLPFAFDILATSFMYGSKTFINHTQGIPDFFKQSFPEGFTWERVTTYEDGGVLTATQDTSLQDGCLIYNVKIRGVNFPSNGPVMQKKTLGWEASTETLYPADGGLEGRADMALKLVGGGHLICNLKTTYRSKKPAKNLKMPGVYYVDRRLERIKEADKETYVEQHEVAVARYCDLPSKLGHR* |

bioRxiv preprint doi: https://doi.org/10.1101/2024.08.08.606661; this version posted July 19, 2025. The copyright holder for this preprint (which was not certified by peer review) is the author/funder, who has granted bioRxiv a license to display the preprint in perpetuity. It is made available under aCC-BY 4.0 International license.

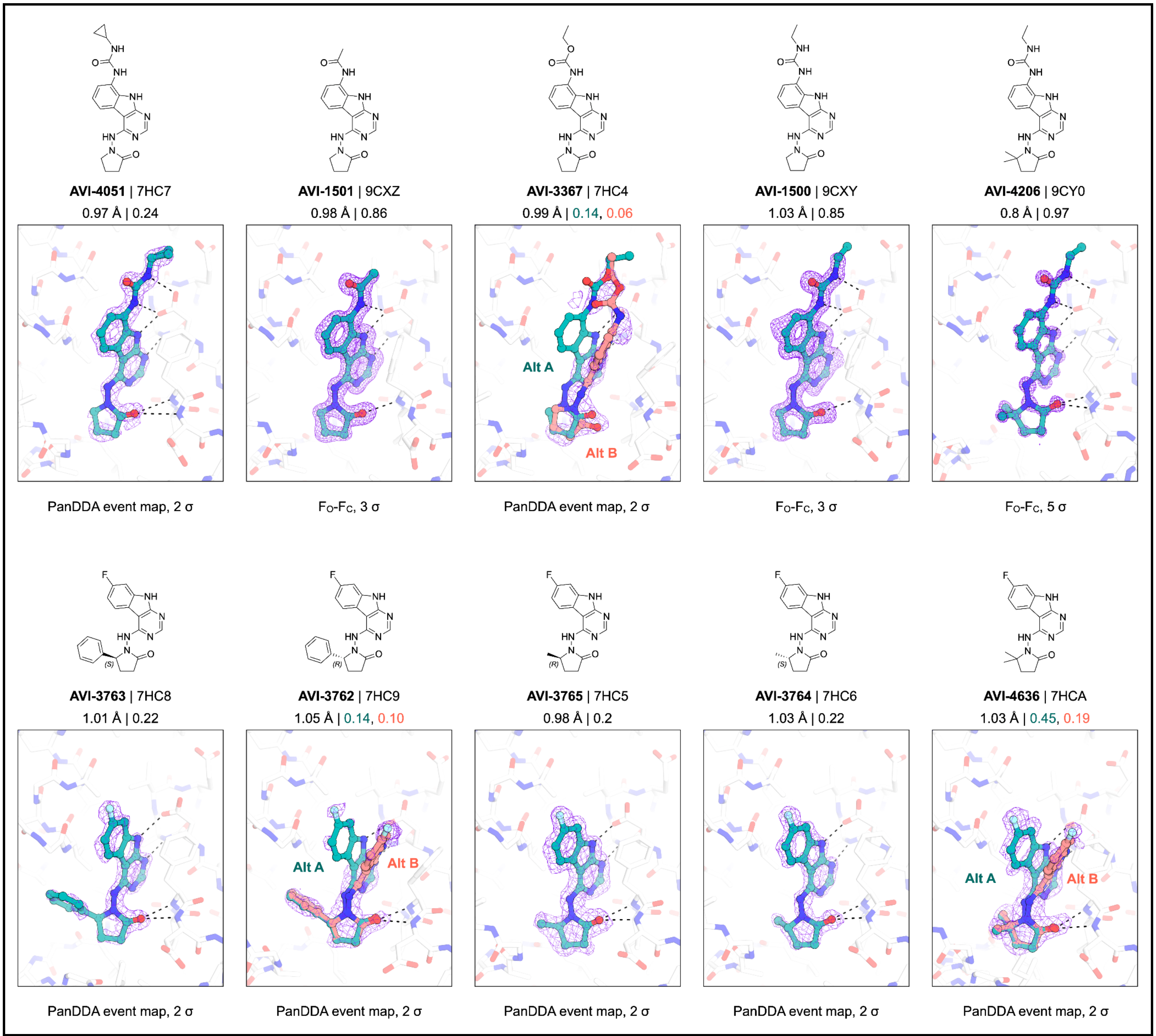


**Supplementary Figure1** - X-ray density for ligand modeling. Ligands were modeled using either traditional $F_O$-$F_C$ electron density maps (AVI-1500, AVI-1501, AVI-4206) or PanDDA event maps (AVI-4051, AVI-3367, AVI-3763, AVI-3762, AVI-3765, AVI-3764 and AVI-4636). The diffraction resolution and refined occupancy are indicated for each ligand. The occupancy is indicated for each confirmation when multiple ligand poses were modeled.

bioRxiv preprint doi: https://doi.org/10.1101/2024.08.08.606661; this version posted July 19, 2025. The copyright holder for this preprint (which was not certified by peer review) is the author/funder, who has granted bioRxiv a license to display the preprint in perpetuity. It is made available under aCC-BY 4.0 International license.

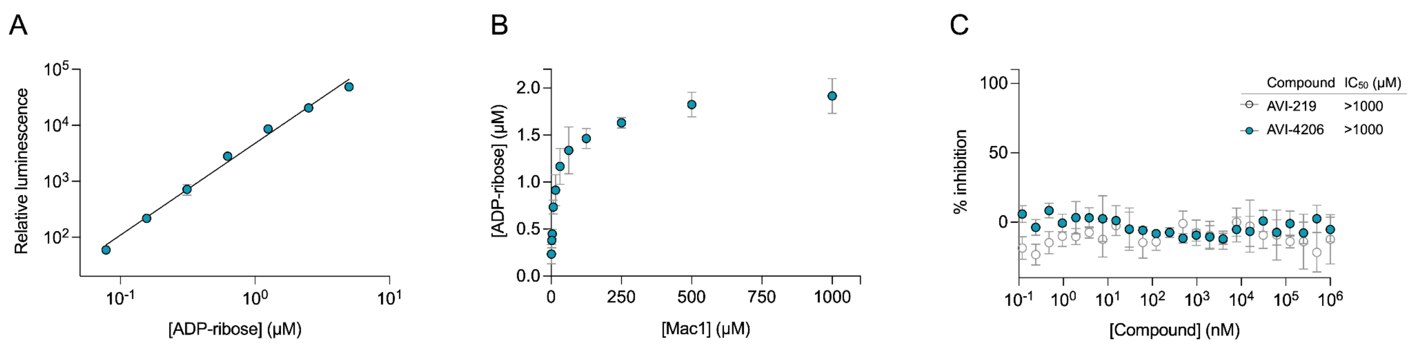


**Supplementary Figure2**. AVI-4206 and AVI-219 inhibition of Mac1 determined using auto-mono-ADP-ribosylated PARP10 as a substrate.

**(A)** Standard curve of ADP-ribose detected using 100 nM NUDT5 and the AMP-Glo assay kit. Data are presented mean ± SD for four technical replicates. Data were fitted with a power function in the form $y = kx^a$ using non-linear regression (gray line).

**(B)** Titration of Mac1 with auto-mono-ADP-ribosylated PARP10. The concentration of PARP10 was 10 μM based on absorbance at 280 nm, but the titration indicated that the concentration of ADP-ribose released by Mac1 was five-fold lower (~2 μM). Data are presented mean ± SD for four technical replicates.

**(C)** Counterscreen of compounds against 100 nM NudT5 with 2 μM ADP-ribose as a substrate. No inhibition was detected up to 1 mM compound. Data are presented mean ± SD for four technical replicates.

bioRxiv preprint doi: https://doi.org/10.1101/2024.08.08.606661; this version posted July 19, 2025. The copyright holder for this preprint (which was not certified by peer review) is the author/funder, who has granted bioRxiv a license to display the preprint in perpetuity. It is made available under aCC-BY 4.0 International license.

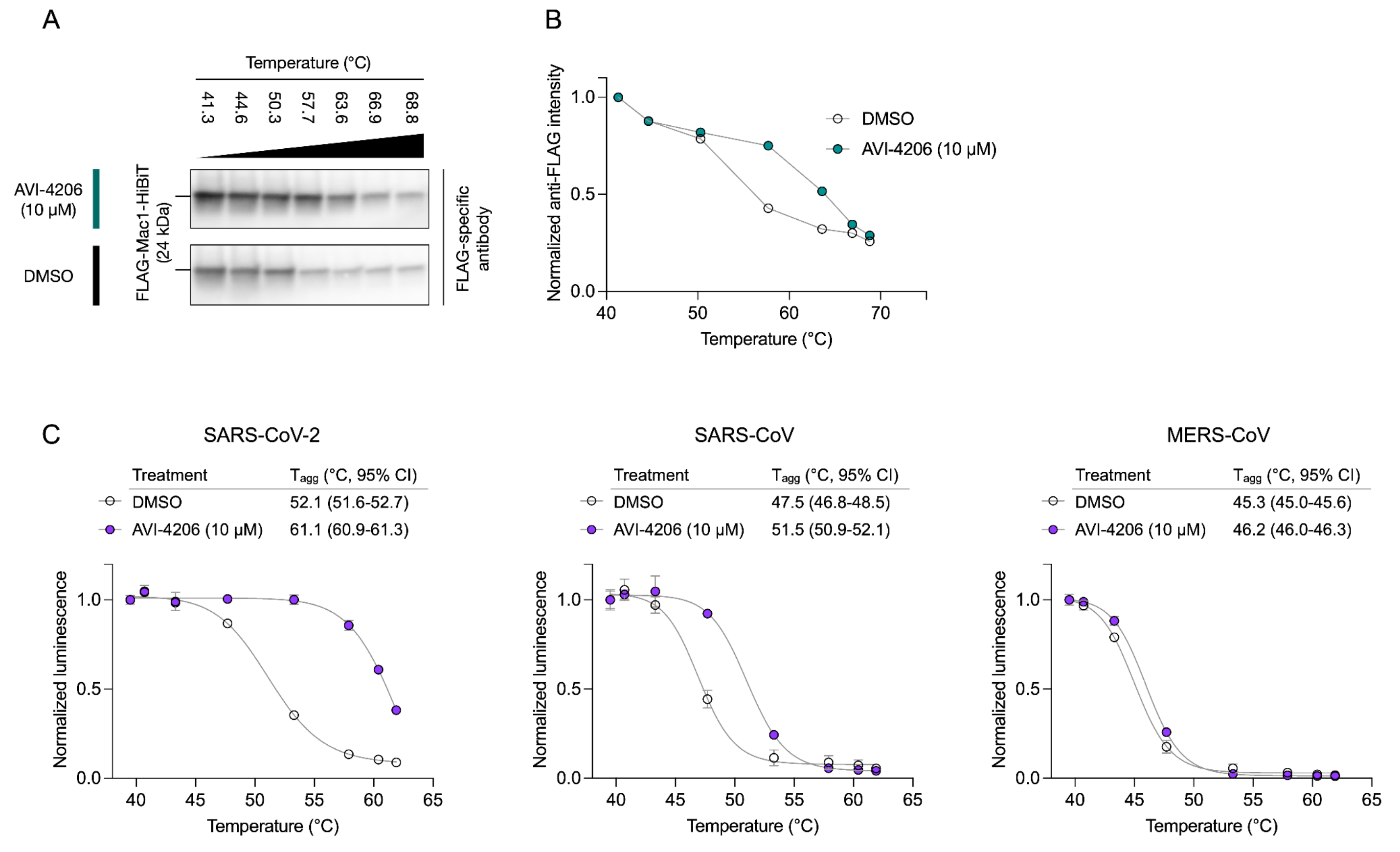


**Supplementary Figure3: AVI-4206 increases thermal stability of Mac1 in cells.**
**(A)** CETSA-WB shows thermal stabilization of FLAG-tagged Mac1 protein after treatment of A549 cells with 10 µM of AVI-4206.
**(B)** Densitometry values were normalized to the lowest temperature for each treatment. Data are presented as a single densitometry measurement.
**(C)** *CETSA-nLuc shows differential stabilization of SARS-CoV2, SARS-CoV and MERS macrodomain proteins in A549 cells treated with 10 µM of AVI-4206. Data are presented as mean ± SD of two technical replicates. Data were fitted with a sigmoidal dose-response equation using non-linear regression (gray line) and the Tagg values are quoted with 95% confidence intervals*

bioRxiv preprint doi: https://doi.org/10.1101/2024.08.08.606661; this version posted July 19, 2025. The copyright holder for this preprint (which was not certified by peer review) is the author/funder, who has granted bioRxiv a license to display the preprint in perpetuity. It is made available under aCC-BY 4.0 International license.

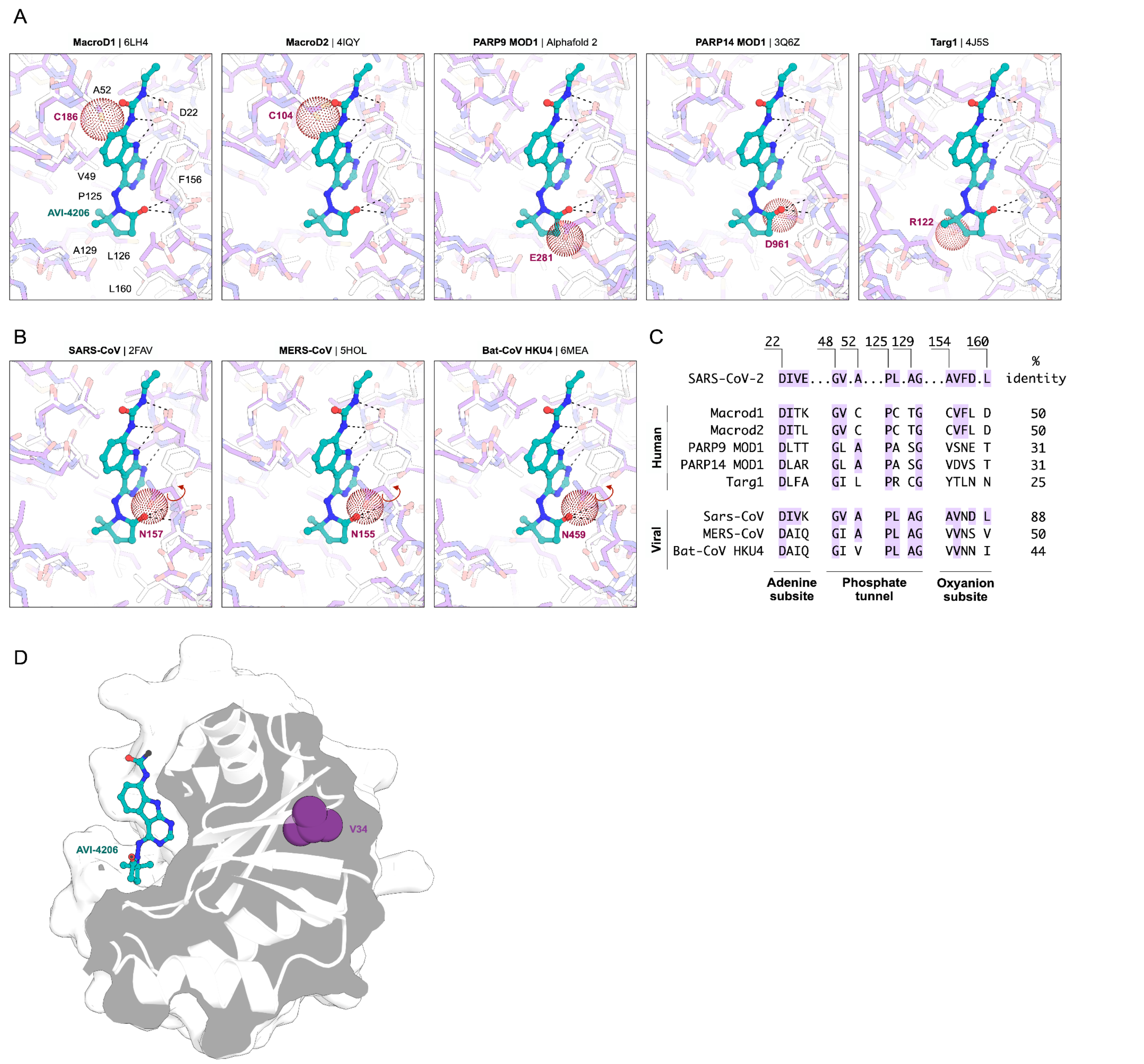


**Supplementary Figure4**: **Alignment of AVI-4206 bound SARS-CoV-2 Mac1 with diverse Macrodomains suggests the origin of AVI-4206 selectivity**. (**A**) For human and (**B**) viral macrodomains, AVI-4206 is shown with teal spheres/sticks, SARS-CoV-2 Mac1 is shown with white sticks and the macrodomains being compared are shown with purple sticks. Selected Mac1 residues are labeled with black text, and the most important clashing human/viral residue is labeled and shown with red dots. For the clashing asparagine residue in the viral macrodomains (position equivalent to Phe156 in SARS-CoV-2 Mac1), rotation of the residue around the χ1 dihedral angle (shown with a red arrow) could relieve the clash and allow AVI-4206 binding. The human/viral macrodomain structures compared are bound to ADP-ribose, except for PARP9 MOD1, where no experimental structure is available and the Alphafold 2 model is shown. (**C**) The SARS-CoV-2 Mac1 sequence was aligned to human/viral macrodomains shown in (**A**) and (**B**) for SARS-CoV-2 Mac1 residues within 5 Å of AVI-4206. The SARS-CoV-2 residue numbering and the % identity for each sequence compared to SARS-CoV-2 Mac1 is indicated.

bioRxiv preprint doi: https://doi.org/10.1101/2024.08.08.606661; this version posted July 19, 2025. The copyright holder for this preprint (which was not certified by peer review) is the author/funder, who has granted bioRxiv a license to display the preprint in perpetuity. It is made available under aCC-BY 4.0 International license.

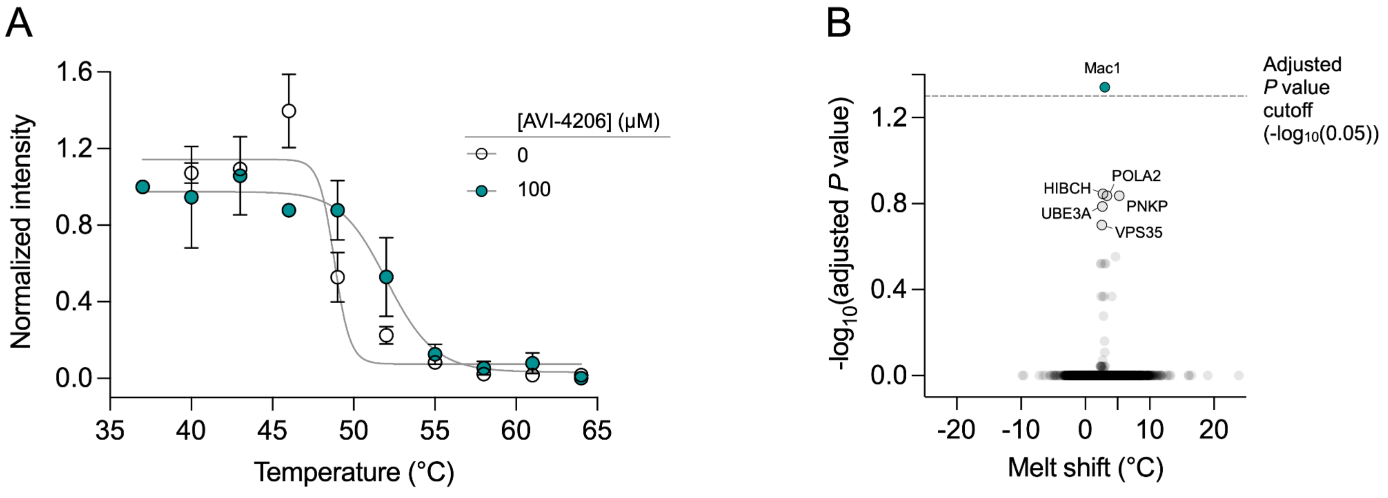


**Supplementary Figure5**: **Thermal proteome profiling in A549 cellular lysates.**
**(A)** Melting curve for Mac1 in A549 lysates treated in duplicate with either DMSO or 100 μM of AVI-4206. Data were normalized to the mean intensity at 37°C. Data were fitted with a sigmoidal dose-response equation using non-linear regression (gray line).
**(B)** Volcano plot of the statistical significance and degree of melting temperature shift for all proteins with high quality melting curves (n= 3,446 proteins). Teal circles indicate proteins with a statistically significant shift in melting temperature (adjusted *P* value < 0.05). The highest non-significant proteins are labeled and do not have obvious functional overlap with macrodomains.

bioRxiv preprint doi: https://doi.org/10.1101/2024.08.08.606661; this version posted July 19, 2025. The copyright holder for this preprint (which was not certified by peer review) is the author/funder, who has granted bioRxiv a license to display the preprint in perpetuity. It is made available under aCC-BY 4.0 International license.

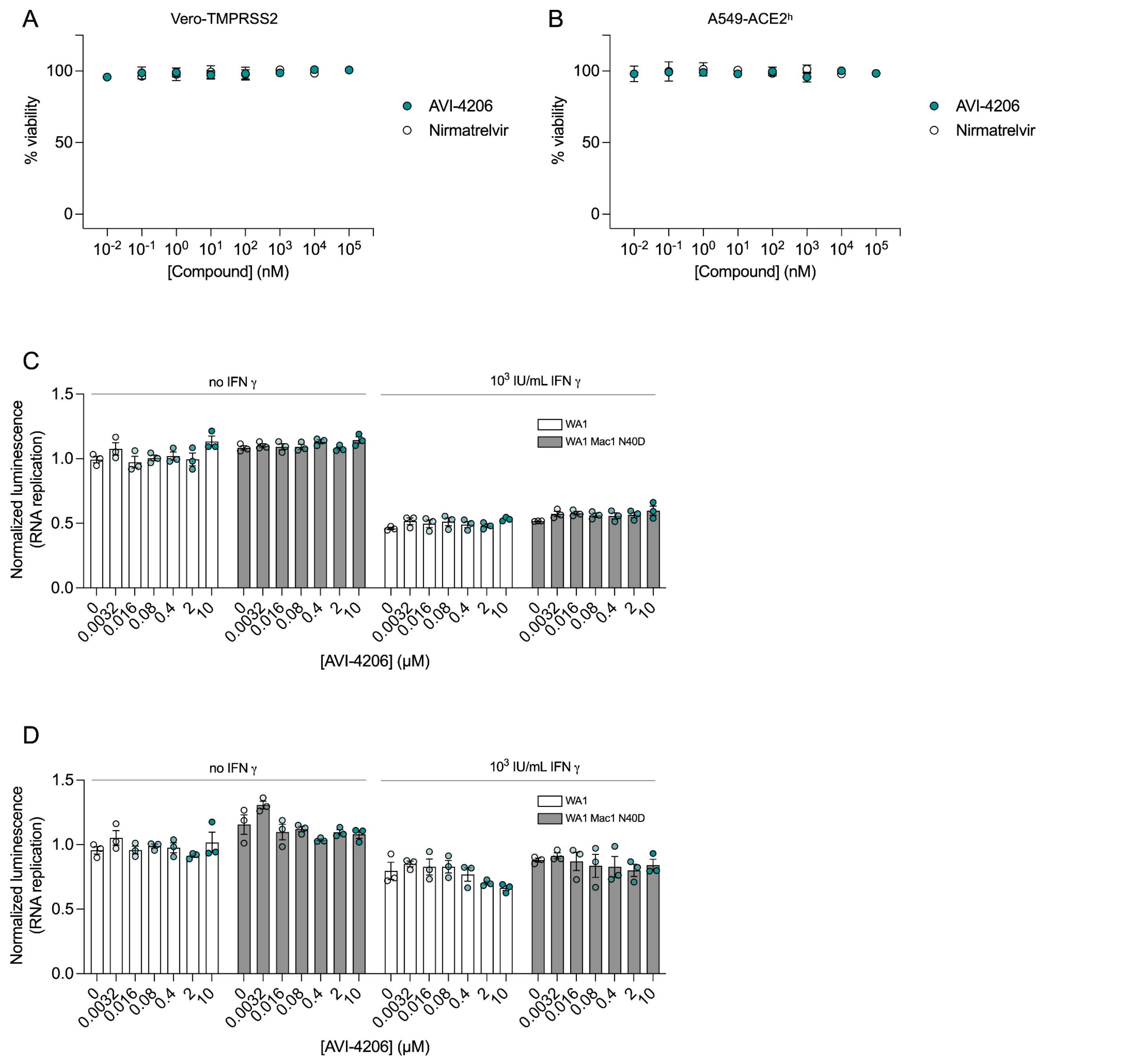


**Supplementary Figure6: AVI-4206 has limited antiviral efficacy and no cytotoxicity in cellular models of infection.**

**(A and B)** Drug cytotoxicity of AVI-4206 in Vero-TMPRSS2 (A) and A549 ACE2$^h$ (B) was measured using the CellTiter-Glo® viability assay. Graphs represent the mean ± SD of three biological replicates each conducted in triplicate.

**(C and D)** Luciferase readout of VAT (C) and A549 ACE2$^h$ (D) cells infected with WA1 or WA1 Mac1 N40D replicons and treated with or without AVI-4206 and IFN-γ at indicated concentrations. Results are plotted as normalized mean ± SD luciferase values of a representative biological experiment containing three technical replicates.

bioRxiv preprint doi: https://doi.org/10.1101/2024.08.08.606661; this version posted July 19, 2025. The copyright holder for this preprint (which was not certified by peer review) is the author/funder, who has granted bioRxiv a license to display the preprint in perpetuity. It is made available under aCC-BY 4.0 International license.

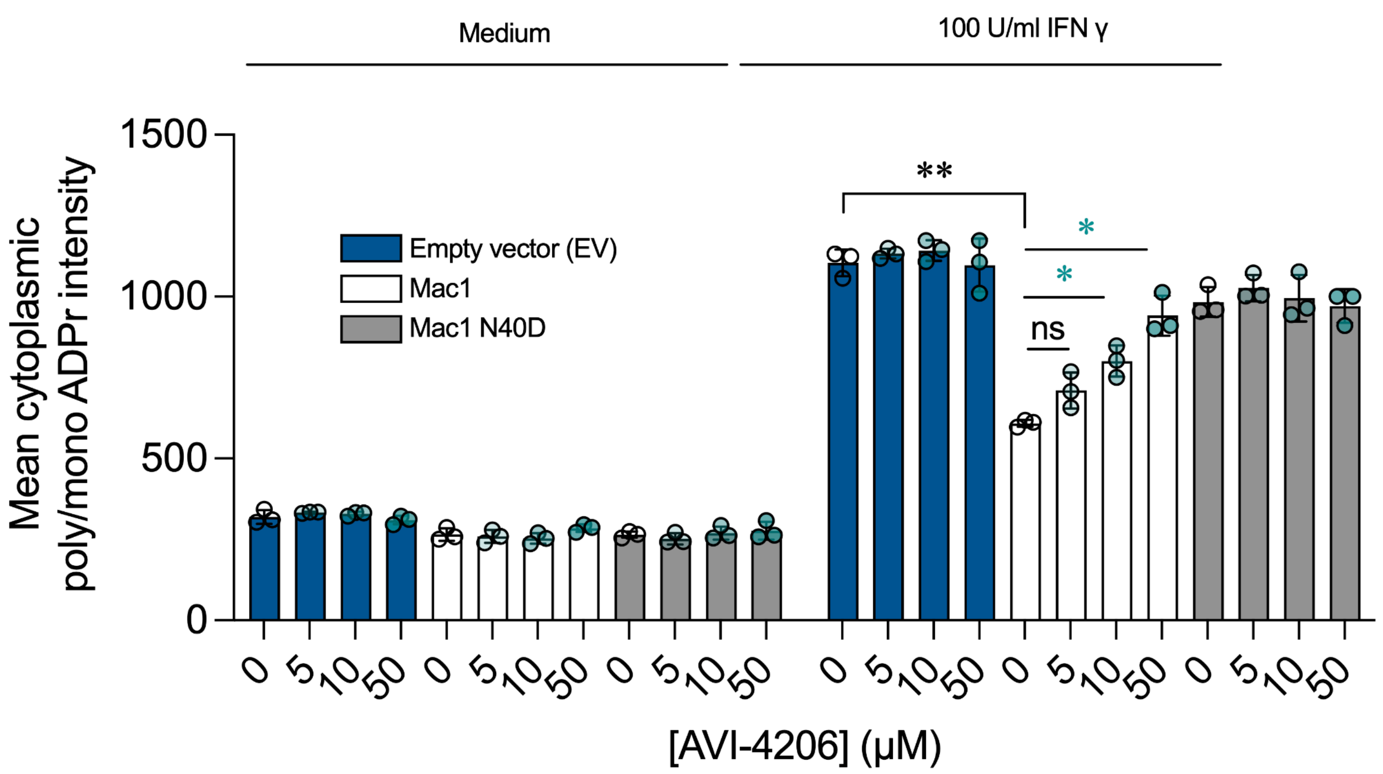


**Supplementary Figure7**. **Relative mean cytoplasmic poly/mono ADPr intensity of A549 cells stably expressing Mac1 and Mac1-N40D treated with IFN-γ and/or AVI-4206**. DMSO-treated cells are shown as vehicle control. Poly/mono ADPr signal comes from Poly/Mono-ADP Ribose (E6F6A) Rabbit mAb (CST, 83732S) staining. Data shown as mean values ± SD; At least 10000 cells were analyzed each group, from triplicate wells. Two-tailed student's t-test were used to compare ADPr intensity levels of each treatment. *, $p < 0.05$; **, $p < 0.01$;

bioRxiv preprint doi: https://doi.org/10.1101/2024.08.08.606661; this version posted July 19, 2025. The copyright holder for this preprint (which was not certified by peer review) is the author/funder, who has granted bioRxiv a license to display the preprint in perpetuity. It is made available under aCC-BY 4.0 International license.

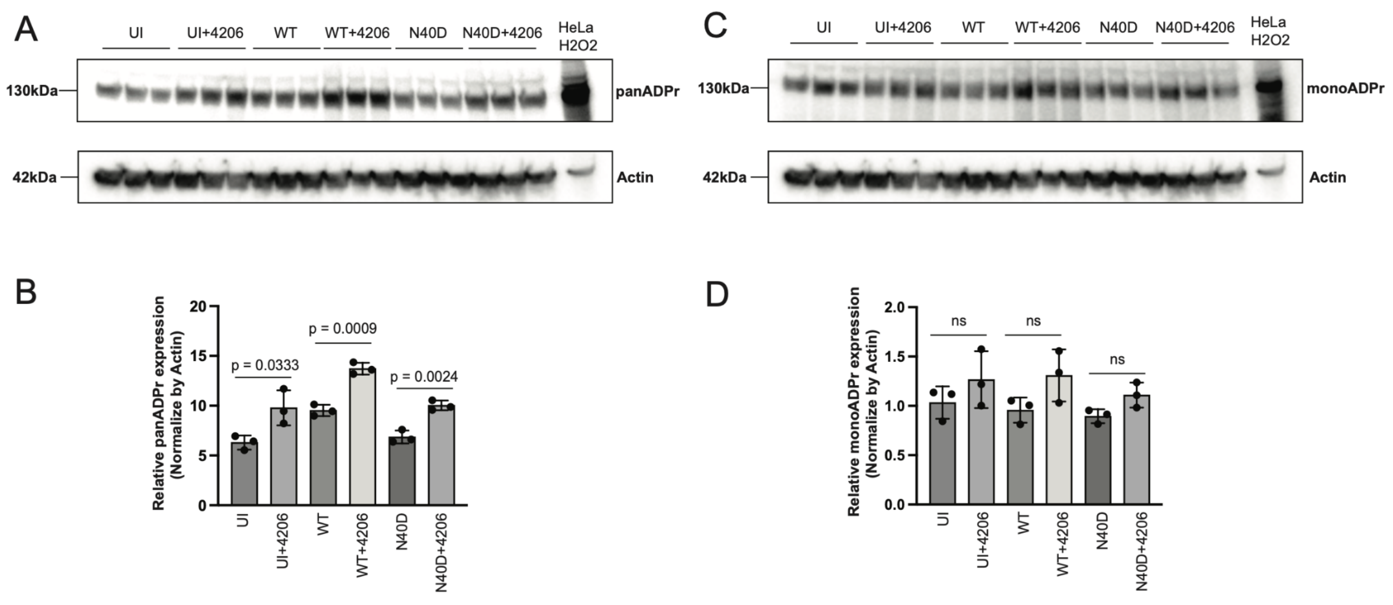


**Supplementary Figure8**: **ADP-ribosylation profiling during infection by Western Blot.** (A) Immunoblot showing pan-ADP-ribose (panADPr) levels in Calu-3 cells under indicated infection conditions. UI = uninfected cells, WT = cells infected with WA1, N40D = cells infected with WA1 NSP3 Mac1 N40D, with or without 100 μM AVI-4206 treatment. HeLa cells treated with $H_2O_2$ were included as a positive control for the ADPr signal. Actin serves as a loading control. (B) Densitometric analysis of panADPr levels normalized to actin. Data are presented as mean ± SD (n = 3), with p-values indicated. (C) Immunoblot showing mono-ADP-ribose (monoADPr) levels under the same conditions as (A). (D) Densitometric analysis of monoADPr levels normalized to actin. No statistically significant differences (ns) were observed between conditions. Data are shown as mean ± SD (n = 3).

bioRxiv preprint doi: https://doi.org/10.1101/2024.08.08.606661; this version posted July 19, 2025. The copyright holder for this preprint (which was not certified by peer review) is the author/funder, who has granted bioRxiv a license to display the preprint in perpetuity. It is made available under aCC-BY 4.0 International license.

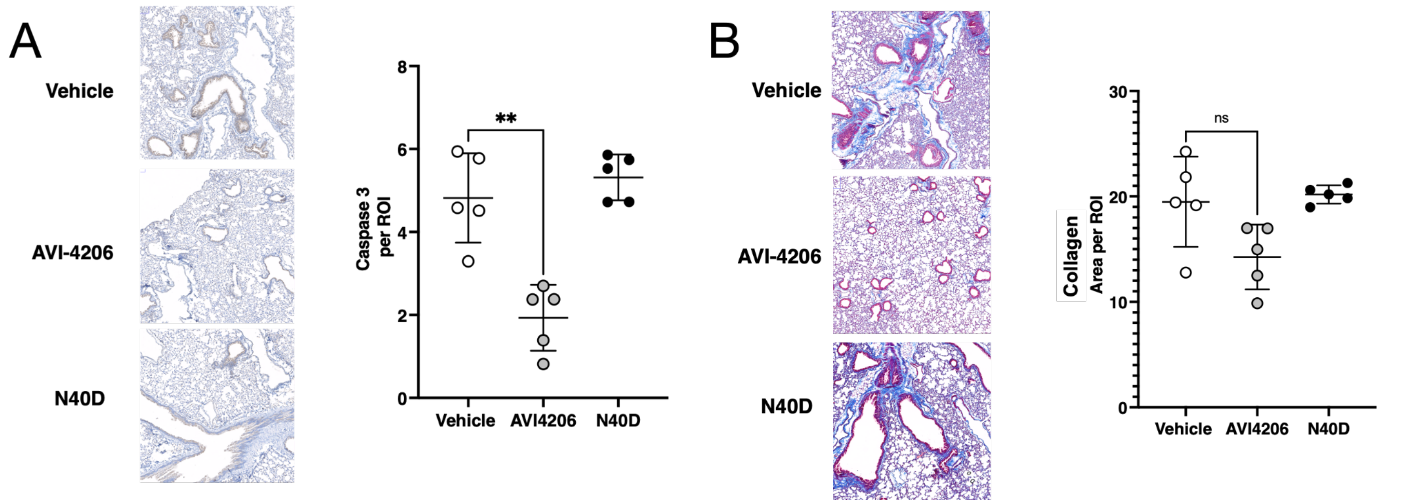


**Supplementary Figure9 Lung Histology**: We examined determinants of tissue damage by either (A) caspase 3 staining for apoptosis or (B) Masson's Trichrome stain for collagen deposition and pulmonary fibrosis. In the lungs of AVI-4206 treated animals, apoptosis is significantly reduced compared to the lungs of the vehicle cohort. While collagen deposition in the lungs of AVI-4206 treated animals is trending lower, the result is not significant. There is no difference in pathology between the N40D cohort and vehicle-treated cohort with these markers. This could suggest that AVI-4206 provides an additional mechanism that results in protection.

bioRxiv preprint doi: https://doi.org/10.1101/2024.08.08.606661; this version posted July 19, 2025. The copyright holder for this preprint (which was not certified by peer review) is the author/funder, who has granted bioRxiv a license to display the preprint in perpetuity. It is made available under aCC-BY 4.0 International license.

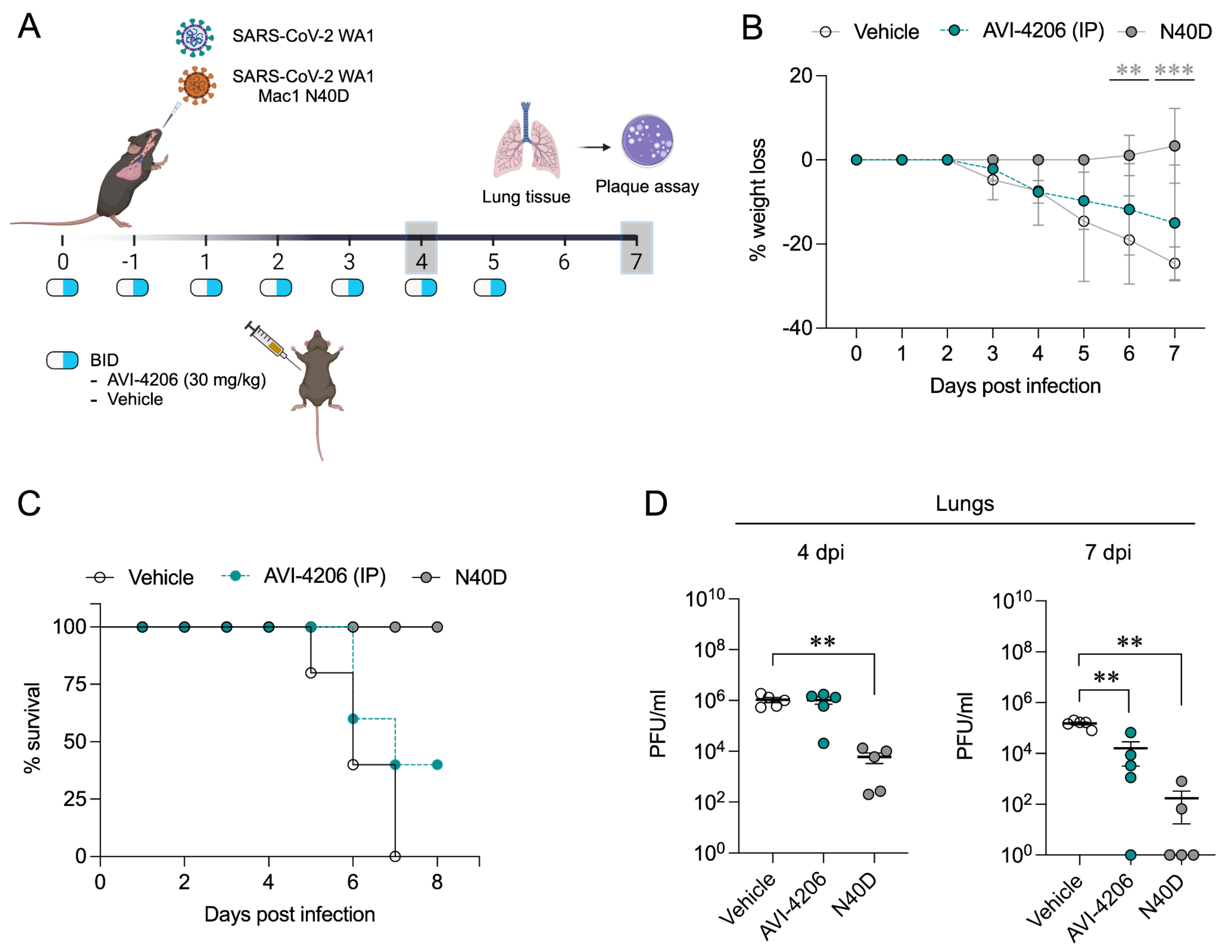


**Supplementary Figure10**: **Lower dose AVI-4206 reduces viral replication and increases survival *in vivo*.**
**(A)** K18-hACE2 mice were intranasally infected SARS-CoV-2 WA1 or SARS-CoV-2 WA1 Mac1 N40D mutant. Mice were treated as indicated with AVI-4206 (BID, 30 mg/kg) or vehicle. Each group was composed of n=10 mice (5 mice per time point).
**(B)** The percent body weight loss is presented as mean ± SD. **, $P < 0.01$; ***, $P < 0.001$ by two-tailed Student's t-test relative to the vehicle control at each timepoint.

**(C)** Survival curve based on the percent body weight loss humane endpoint.
**(D)** Viral load in the lung at indicated time points is presented as mean ± s.e.m. **, $P < 0.01$ by Mann Whitney's test relative to the vehicle control.

bioRxiv preprint doi: https://doi.org/10.1101/2024.08.08.606661; this version posted July 19, 2025. The copyright holder for this preprint (which was not certified by peer review) is the author/funder, who has granted bioRxiv a license to display the preprint in perpetuity. It is made available under aCC-BY 4.0 International license.

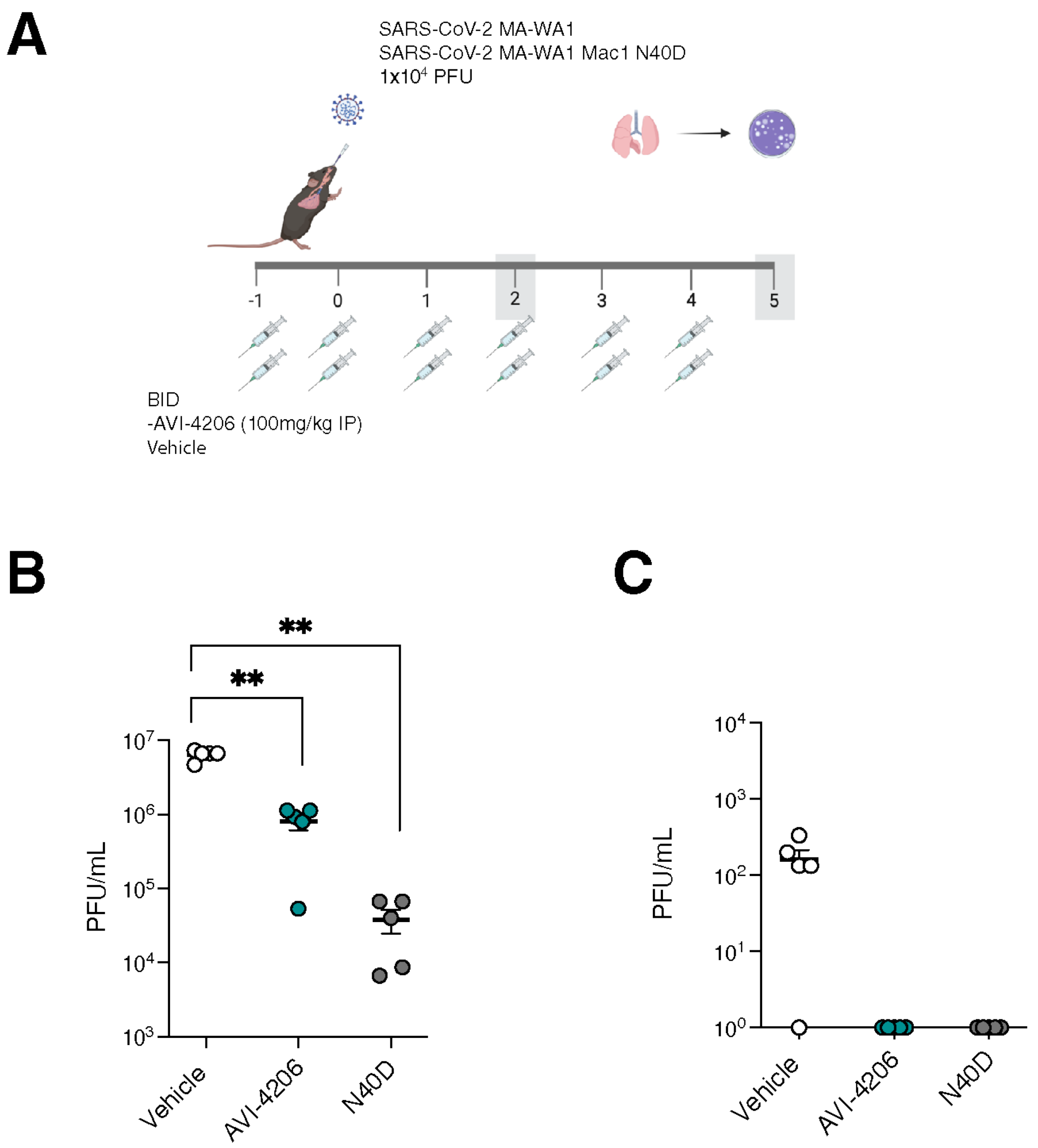


**Supplementary Figure11: AVI-4206 suppresses replication of mouse-adapted SARS-CoV-2 in wild-type mice.**

(A) Wild-type mice were intranasally infected with SARS-CoV-2 and treated with AVI-4206, AVI-6451, or vehicle (n = 10 per group). Mice infected with the WA1 N40D mutant served as a positive control (n = 10). Lung tissues were collected at designated time points for viral titer analysis using a plaque assay.
(B) (C) Viral Load in the Lungs of Wild-Type Mice Treated with AVI-4206 (B, day2; C, day5).**, P < 0.01 by Mann Whitney's test relative to the vehicle control.
