# Supplementary material for: The Mac1 ADP-ribosylhydrolase is a Therapeutic Target for SARS-CoV-2
Source: bioRxiv. 2024 Aug 9:2024.08.08.606661. Preprint. [Version 1] doi: 10.1101/2024.08.08.606661 (PMC11326214; doi:10.1101/2024.08.08.606661)
Supplement: Supplementary file 1 [file NIHPP2024.08.08.606661v1-supplement-1.pdf]

bioRxiv preprint doi: https://doi.org/10.1101/2024.08.08.606661; this version posted August 9, 2024. The copyright holder for this preprint (which was not certified by peer review) is the author/funder, who has granted bioRxiv a license to display the preprint in perpetuity. It is made available under aCC-BY 4.0 International license.

Supplementary information

Supplementary Table 1 - X-ray data collection and refinement deposition statistics.

	Ligand	AVI-1500	AVI-1501	AVI-3367	AVI-3765	AVI-3764	AVI-4051	AVI-3763	AVI-3762	AVI-4636	AVI-4206
	PDB code	9CXY	9CXZ	7HC4	7HC5	7HC6	7HC7	7HC8	7HC9	7HCA	9CY0
	Beam line	ALS 8.3.1	ALS 8.3.1	ALS 8.3.1	ALS 8.3.1	ALS 8.3.1	ALS 8.3.1	SSRL 9-2	SSRL 9-2	ALS 8.3.1	ALS 8.3.1
	Wavelength (Å)	0.88557	0.88557	0.88557	0.88557	0.88557	0.88557	0.88557	0.88557	0.88557	0.77487
	Resolution range (Å)	39.9-1.03 (1.07-1.03)	44.5-1.02 (1.06 -1.02)	39.7-1.00 (1.04 -1.00)	44.4-0.98 (1.02-0.98)	44.5-1.03 (1.07-1.03)	39.7-0.99 (1.03-0.99)	39.7-1.01 (1.05-1.01)	39.7-1.05 (1.09-1.05)	44.47-1.03 (1.07-1.03)	33.7-0.80 (0.83-0.80)
	Space group	$P4_3$	$P4_3$	$P4_3$	$P4_3$	$P4_3$	$P4_3$	$P4_3$	$P4_3$	$P4_3$	P1
	Unit cell (α β γ, a b c)	88.8 88.8 39.5 90 90 90	88.9 88.9 39.4 90 90 90	88.8 88.8 39.5 90 90 90	88.8 88.8 39.5 90 90 90	88.8 88.8 39.6 90 90 90	88.8 88.8 39.4 90 90 90	88.8 88.8 39.6 90 90 90	88.8 88.8 39.6 9 0 90 90	88.9 88.9 39.3 90 90 90	30.3 39.8 64.4 89.9 77.8 90.1
	Total reflections	925861 (88714)	1019499 (92950)	1052667 (79477)	1095419 (68284)	981382 (94605)	1075242 (76799)	1049402 (101219)	934650 (89428)	996315 (94266)	420519 (10537)
	Unique reflections	151957 (14738)	156086 (15234)	165160 (15511)	175278 (16099)	152839 (14932)	169798 (16087)	161313 (15833)	142525 (13787)	151748 (14847)	235488 (6157)
	Multiplicity	6.1 (5.9)	6.5 (6.1)	6.4 (5.1)	6.2 (4.1)	6.4 (6.3)	6.3 (4.7)	6.5 (6.3)	6.6 (6.4)	6.6 (6.3)	1.8 (1.7)
	Completeness (%)	99.51 (96.98)	99.59 (97.52)	99.30 (93.65)	99.13 (91.70)	99.77 (98.28)	99.44 (94.83)	99.70 (98.49)	98.59 (96.15)	99.74 (98.30)	75.89 (19.87)
	Mean I/sigma(I)	13.96 (0.68)	15.02 (1.04)	13.32 (0.77)	11.98 (0.58)	9.03 (0.70)	12.87 (0.79)	12.16 (0.64)	10.78 (0.66)	13.86 (0.67)	13.15 (1.22)
	Wilson B-factor	13.38	12.91	12.4	12.14	11.92	12.15	11.98	12.53	14.12	6.69
	R-merge	0.0459 (2.25)	0.0439 (1.39)	0.0489 (1.38)	0.0546 (1.61)	0.0789 (1.73)	0.0521 (1.34)	0.0589 (2.38)	0.0672 (2.3)	0.0458 (2.24)	0.0334 (0.463)
	R-meas	0.0503 (2.47)	0.0477 (1.52)	0.0532 (1.54)	0.0594 (1.85)	0.0860 (1.88)	0.0567 (1.5)	0.0640 (2.60)	0.0730 (2.51)	0.0498 (2.44)	0.0472 (0.655)
	R-pim	0.0203 (0.995)	0.0184 (0.607)	0.0207 (0.665)	0.0233 (0.879)	0.0337 (0.751)	0.0222 (0.669)	0.0249 (1.03)	0.0282 (0.979)	0.0193 (0.961)	0.0334 (0.463)
	$CC_{1/2}$	0.999 (0.463)	0.999 (0.709)	0.999 (0.547)	0.999 (0.405)	0.998 (0.537)	0.999 (0.582)	0.999 (0.466)	0.999 (0.484)	0.999 (0.424)	0.997 (0.643)
	CC*	1 (0.795)	1 (0.911)	1 (0.841)	1 (0.759)	1 (0.836)	1 (0.858)	1 (0.797)	1 (0.808)	1 (0.772)	0.999 (0.885)
	Reflections used in refinement	151730 (14738)	156007 (15234)	164947 (15512)	174746 (16099)	152688 (14932)	169673 (16087)	161090 (15833)	142225 (13787)	151557 (14847)	235328 (6157)
	Reflections used for R-free	7357 (789)	7525 (740)	7958 (711)	8434 (781)	7391 (787)	8190 (751)	7762 (741)	6888 (690)	7340 (790)	11753 (303)
	R-work	0.1436 (0.4057)	0.1432 (0.3464)	0.1416 (0.4193)	0.1434 (0.4132)	0.1439 (0.4514)	0.1423 (0.3854)	0.1502 (0.4026)	0.1549 (0.5311)	0.1552 (0.3774)	0.1620 (0.2952)
	R-free	0.1609 (0.4006)	0.1594 (0.3372)	0.1583 (0.4254)	0.1587 (0.3953)	0.1595 (0.4464)	0.1580 (0.3611)	0.1686 (0.3973)	0.1773 (0.5333)	0.1735 (0.3805)	0.1884 (0.2959)
	CC(work)	0.977 (0.759)	0.975 (0.874)	0.975 (0.807)	0.975 (0.741)	0.975 (0.823)	0.974 (0.825)	0.973 (0.785)	0.974 (0.716)	0.972 (0.737)	0.954 (0.799)
	CC(free)	0.971 (0.779)	0.974 (0.881)	0.967 (0.823)	0.965 (0.777)	0.966 (0.750)	0.969 (0.847)	0.963 (0.779)	0.964 (0.677)	0.964 (0.753)	0.933 (0.763)
Number of atoms	Number of non-hydrogen atoms	3082	3046	3759	3756	3658	3675	3488	3811	4148	6135
	macromolecules	2609	2615	3260	3253	3162	3177	2992	3315	3644	5240
	ligands	35	31	45	37	37	47	43	43	39	204
	solvent	446	406	472	480	473	470	469	469	481	783
	Protein residues	338	338	337	336	336	336	336	337	337	336
RMS	Bonds (Å)	0.175	0.144	0.038	0.006	0.006	0.006	0.006	0.11	0.056	0.317
	Angles) (°)	3.98	3.8	1.3	0.98	0.96	0.95	0.98	2.27	1.71	5.69
Ramach-andran	Favored (%)	99.1	98.8	99.4	99.4	99.4	99.4	99.4	99.4	99.1	98.8
	Allowed (%)	0.9	1.2	0.6	0.6	0.6	0.6	0.6	0.6	0.9	1.2
	Outliers (%)	0	0	0	0	0	0	0	0	0	0
	Rotamer outliers (%)	0	0	2.54	1.12	0.87	0.86	1.81	1.39	0.76	0.69
	Clashscore	0.76	1.51	2.13	2.28	2.03	2.65	2.15	4.49	2.85	3.11
B-factor ($Å^2$)	Average	21.5	20.92	19.02	18.79	18.04	19.08	19.44	19.55	22.95	9.88
	Macromolecules	19.43	19.14	17.11	16.99	16.18	17.19	17.55	17.73	20.99	8.95
	Ligands	18.01	16.17	17.48	12.72	13.56	17.7	17.35	18.26	17.1	6.82
	Solvent	33.82	32.71	32.34	31.27	30.7	31.88	31.62	32.52	38.09	16.58

bioRxiv preprint doi: https://doi.org/10.1101/2024.08.08.606661; this version posted August 9, 2024. The copyright holder for this preprint (which was not certified by peer review) is the author/funder, who has granted bioRxiv a license to display the preprint in perpetuity. It is made available under aCC-BY 4.0 International license.

Supplementary Table 2 - Eurofins scanEDGE kinase assay shows no inhibition greater than >35% at 10 μM across a panel of diverse kinases

Compound Name	Entrez Gene Symbol	Percent Control
AVI-4206	ABL1	74
AVI-4206	ABL1	95
AVI-4206	ABL1	67
AVI-4206	ABL1	78
AVI-4206	ACVR1B	91
AVI-4206	CABC1	100
AVI-4206	AKT1	80
AVI-4206	AKT2	100
AVI-4206	ALK	69
AVI-4206	AURKA	95
AVI-4206	AURKB	77
AVI-4206	AXL	84
AVI-4206	BMPR2	92
AVI-4206	BRAF	70
AVI-4206	BRAF	99
AVI-4206	BTK	100
AVI-4206	CDK19	99
AVI-4206	CDK2	86
AVI-4206	CDK3	100
AVI-4206	CDK7	99
AVI-4206	CDK9	100
AVI-4206	CHEK1	90

bioRxiv preprint doi: https://doi.org/10.1101/2024.08.08.606661; this version posted August 9, 2024. The copyright holder for this preprint (which was not certified by peer review) is the author/funder, who has granted bioRxiv a license to display the preprint in perpetuity. It is made available under aCC-BY 4.0 International license.

AVI-4206	CSF1R	84
AVI-4206	CSNK1D	88
AVI-4206	CSNK1G2	97
AVI-4206	DCLK1	91
AVI-4206	DYRK1B	99
AVI-4206	EGFR	91
AVI-4206	EGFR	95
AVI-4206	EPHA2	100
AVI-4206	ERBB2	91
AVI-4206	ERBB4	100
AVI-4206	MAPK3	98
AVI-4206	PTK2	89
AVI-4206	FGFR2	100
AVI-4206	FGFR3	97
AVI-4206	FLT3	97
AVI-4206	GSK3B	81
AVI-4206	IGF1R	100
AVI-4206	CHUK	82
AVI-4206	IKBKB	93
AVI-4206	INSR	68
AVI-4206	JAK2	94
AVI-4206	JAK3	93
AVI-4206	MAPK8	88
AVI-4206	MAPK9	73

bioRxiv preprint doi: https://doi.org/10.1101/2024.08.08.606661; this version posted August 9, 2024. The copyright holder for this preprint (which was not certified by peer review) is the author/funder, who has granted bioRxiv a license to display the preprint in perpetuity. It is made available under aCC-BY 4.0 International license.

AVI-4206	MAPK10	88
AVI-4206	KIT	100
AVI-4206	KIT	100
AVI-4206	KIT	89
AVI-4206	STK11	39
AVI-4206	MAP3K4	96
AVI-4206	MAPKAPK2	85
AVI-4206	MARK3	94
AVI-4206	MAP2K1	55
AVI-4206	MAP2K2	60
AVI-4206	MET	98
AVI-4206	MKNK1	68
AVI-4206	MKNK2	70
AVI-4206	MAP3K9	100
AVI-4206	MAPK14	99
AVI-4206	MAPK11	91
AVI-4206	PAK1	100
AVI-4206	PAK2	88
AVI-4206	PAK4	100
AVI-4206	CDK16	83
AVI-4206	PDGFRA	41
AVI-4206	PDGFRB	91
AVI-4206	PDPK1	85
AVI-4206	PIK3C2B	100

bioRxiv preprint doi: https://doi.org/10.1101/2024.08.08.606661; this version posted August 9, 2024. The copyright holder for this preprint (which was not certified by peer review) is the author/funder, who has granted bioRxiv a license to display the preprint in perpetuity. It is made available under aCC-BY 4.0 International license.

AVI-4206	PIK3CA	100
AVI-4206	PIK3CG	100
AVI-4206	PIM1	100
AVI-4206	PIM2	86
AVI-4206	PIM3	100
AVI-4206	PRKACA	84
AVI-4206	PLK1	86
AVI-4206	PLK3	90
AVI-4206	PLK4	91
AVI-4206	PRKCE	76
AVI-4206	RAF1	95
AVI-4206	RET	100
AVI-4206	RIOK2	61
AVI-4206	ROCK2	74
AVI-4206	RPS6KA3	67
AVI-4206	NUAK2	68
AVI-4206	SRC	82
AVI-4206	SRPK3	92
AVI-4206	TGFBR1	84
AVI-4206	TEK	100
AVI-4206	NTRK1	99
AVI-4206	TSSK1B	100
AVI-4206	TYK2	100
AVI-4206	ULK2	100

bioRxiv preprint doi: https://doi.org/10.1101/2024.08.08.606661; this version posted August 9, 2024. The copyright holder for this preprint (which was not certified by peer review) is the author/funder, who has granted bioRxiv a license to display the preprint in perpetuity. It is made available under aCC-BY 4.0 International license.

AVI-4206	KDR	45
AVI-4206	STK32C	85
AVI-4206	ZAP70	91

Supplementary Table 3 Pharmacokinetic parameters for AVI-4206 following IV (10 mg/kg), PO (50 mg/kg), and IP (100 mg/kg) doses in male CD1 mice (n = 3 per group).

IV (10 mg/kg)			PO (50 mg/kg)			IP (100 mg/kg)		
parameter	units	value	parameter	units	value	parameter	units	value
CL	ml/min/kg	69.5	T_{max}	hr	1.0	T_{max}	hr	0.250
V_{ss}	l/kg	1.32	C_{max}	ng/ml	94.0	C_{max}	ng/ml	38067
$T_{1/2}$	hr	0.833	$T_{1/2}$	hr	4.69	$T_{1/2}$	hr	3.17
AUC_{last}	hr*ng/ml	2396	AUC_{last}	hr*ng/ml	433	AUC_{last}	hr*ng/ml	40998
AUC_{INF}	hr*ng/ml	2399	AUC_{INF}	hr*ng/ml	442	AUC_{INF}	hr*ng/ml	41003
MRT_{INF}	hr	0.316	F	%	3.69	F	%	171

Liver blood flow (mouse) = 120 ml/min/kg.

Supplementary Table 4 - ADMET panel shows no antagonist response greater than >15% at 10 µM.

**Assay**	**Ligand or Substrate**	**Antagonist Response at 10 µM**
Cholinesterase, Acetyl, ACES	Acetylthiocholine	4.1
Peptidase Bleomycin Hydrolase (BLMH)	Met-AMC	-0.4
Peptidase, CAN1 (CANPL1, Calpain-1)	Casein-FITC	5
Peptidase, CTSB (Cathepsin B)	Boc-Leu-Arg-Arg-AMC	-17.5
Peptidase, CTSG (Cathepsin G)	Suc-Ala-Ala-Pro-Phe-AMC	-3.2
Peptidase, CTSH (Cathepsin H)	L-Arg-AMC	-13.7
Peptidase, CTSK (Cathepsin K)	Z-Phe-Arg-AMC	-5
Peptidase, CTSL (Cathepsin L)	Z-Phe-Arg-AMC	2.6
Peptidase, CTSS (Cathepsin S)	Z-Leu-Arg-AMC	-50.7
Peptidase, CTSL2 (Cathepsin L2)	Z-Leu-Arg-AMC	-1.2
Peptidase, CTSZ (Cathepsin Z)	Mca-Arg-Pro-Pro-Gly-Phe-Ser-Ala-Phe-Lys(Dnp)-OH	-7.1
Peptidase, Chymase	Suc-Ala-Ala-Pro-Phe-AMC	-5.1
Peptidase, Chymotrypsin	Suc-Ala-Ala-Pro-Phe-AMC	-0.7
Peptidase, Factor VIIa	N-CH3-SO2-D-Phe-Gly-Arg-pNA	-3.3
Peptidase Kallikrein Plasma	Z-FR-AMC	7.9
Peptidase, PLAU (Urokinase)	Z-Gly-Gly-Arg-AMC	11.2
Cyclooxygenase COX-1	Arachidonic acid	0.6
Cyclooxygenase COX-2	Arachidonic Acid	5.7
Monoamine Oxidase MAO-A	Kynuramine	3.3
Phosphodiesterase PDE3A	FAM-cAMP	0.6

bioRxiv preprint doi: https://doi.org/10.1101/2024.08.08.606661; this version posted August 9, 2024. The copyright holder for this preprint (which was not certified by peer review) is the author/funder, who has granted bioRxiv a license to display the preprint in perpetuity. It is made available under aCC-BY 4.0 International license.

Phosphodiesterase PDE4D2	FAM-cAMP	0.8
Peptidase, CASP2 (Caspase 2)	Z-VDVAD-AFC	4.1
Peptidase, CASP4 (Caspase 4)	Ac-LEVD-AFC	-2.9
Peptidase, CASP5 (Caspase 5)	Ac-WEHD-AFC	-6.6
Peptidase, Prolyl Oligopeptidase (POP)	Z-Gly-Pro-AMC	-0.5
Peptidase, Plasmin	H-D-Val-Leu-Lys-pNA	-1.5
Peptidase, Thrombin	Z-Gly-Pro-Arg-AMC	0.4
Peptidase, Tissue Plasminogen Activator (tPA)	N-alpha-Z-D-Arg-Gly-Arg-pNA	1.5
Peptidase, Trypsin	Z-Gly-Pro-Arg-AMC	-1.6
Peptidase, Tryptase	Z-Gly-Pro-Arg-AMC	-0.5
Peptidase, ELA2 (Neutrophil Elastase 2)	N-MeOSuc-Ala-Ala-Pro-Val-pNA	-3
Peptidase, ELA1 (Pancreatic Elastase 1)	N-MeOSuc-Ala-Ala-Pro-Val-pNA	-2.2
Protein Tyrosine Kinase, LCK	Poly(Glu:Tyr)	-1.4
Peptidase, Dipeptidyl Peptidase 4 (DPP4, DPP IV)	GP-AMC	1.3
Peptidase, CASP1 (Caspase 1)	Z-YUAD-AFC	-0.9
Adenosine A2A	[3H]CGS-21680	-1
Adrenergic alpha1A	[3H]Prazosin	2.1
Adrenergic alpha2A	[3H]Rauwolscine	4.9
Adrenergic beta1	[125I]Cyanopindolol	2.4
Adrenergic beta2	[3H]CGP-12177	-10.5
Transporter, Norepinephrine (NET)	[125I]RTI-55	-2.4
Androgen (Testosterone)	[3H]Methyltrienolone	-1.5
Calcium Channel L-Type, Dihydropyridine	[3H]Nitrendipine	-6.2
Cannabinoid CB1	[3H]SR141716A	4.2
Cannabinoid CB2	[3H]WIN-55,212-2	6.7
Cholecystokinin CCK1 (CCKA)	[125I]CCK-8	-6.9
Dopamine D1	[3H]SCH-23390	-12.1
Dopamine D2S	[3H]Spiperone	-15.8
Transporter, Dopamine (DAT)	[125I]RTI-55	6
Endothelin ETA	[125I]Endothelin-1	3
GABAA, Flunitrazepam, Central	[3H]Flunitrazepam	-9.9
Glucocorticoid	[3H]Dexamethasone	6.9
Glutamate, NMDA, Agonism	[3H]CGP-39653	-2.9
Histamine H1	[3H]Pyrilamine	-12.8
Histamine H2	[125I]Aminopotentidine	2.5
Muscarinic M1	[3H]N-Methylscopolamine	-8.1
Muscarinic M2	[3H]N-Methylscopolamine	-0.4
Muscarinic M3	[3H]N-Methylscopolamine	-2.6
Opiate delta1 (OP1, DOP)	[3H]Naltrindole	-3.2

bioRxiv preprint doi: https://doi.org/10.1101/2024.08.08.606661; this version posted August 9, 2024. The copyright holder for this preprint (which was not certified by peer review) is the author/funder, who has granted bioRxiv a license to display the preprint in perpetuity. It is made available under aCC-BY 4.0 International license.

Opiate kappa (OP2, KOP)	[3H]Diprenorphine	6.3
Opiate mu (OP3, MOP)	[3H]Diprenorphine	8.2
Potassium Channel [KA]	[125I]alpha-Dendrotoxin	5.6
Potassium Channel hERG, [3H]Dofetilide	[3H]Dofetilide	-4.7
Serotonin (5-Hydroxytryptamine) 5-HT1A	[3H]8-OH-DPAT	-2.3
Serotonin (5-Hydroxytryptamine) 5-HT1B	[3H]GR125743	2.2
Serotonin (5-Hydroxytryptamine) 5-HT2A	[3H]Ketanserin	8.3
Serotonin (5-Hydroxytryptamine) 5-HT2B	[3H]Lysergic acid diethylamide (LSD)	-4.5
Serotonin (5-Hydroxytryptamine) 5-HT3	[3H]GR-65630	-3.2
Transporter, Serotonin (5-Hydroxytryptamine) (SERT)	[3H]Paroxetine	-2.2
Sodium Channel Nav1.5	[3H]BNZA	0.3
Vasopressin V1A	[125I]PhenylacetylTyr(Me)PheGlnAsnArgProArgTyr	2
Nicotinic Acetylcholine alpha4beta2, Cytisine	[3H]Cytisine	4.7

Supplementary Table 5 HTRF Macrodomain protein sequences

Protein	Sequence
hMacroD2	M**HHHHHH**SSGVDLGTENLYFQSYPSNKKKKVWREEKERLLKMTLEERRKEYLRDYIPLNSILSWKEEMKGKGQNDEENTQETSQVKKSLTEKVSLYRGDITLLEVDAIVNAANASLLGGGGVDGCIHRAAGPCLLAECRNLNGCDTGHAKITCGYDLPAKYVIHTVGPIARGHINGSHKEDLANCYKSSLKLVKENNIRSVAFPCISTGIYGFPNEPAAVIALNTIKEWLAKNHHEVDRIIFCVFLEVDFKIYKKKMNEFFSVDDNNEEEEDVEMKEDSDENGPEEKQSVEEMEEQSQDADGVNTVTVPGPASEEAVEDCKDEDFAKDENITKGGEVTDHSVRDQDHPDGQENDSTKNEIKIETESQSSYMETEELSSNQEDAVIVEQPEVIPLTEDQEEKEGEKAPGEDTPRMPGKSEGSSDLENTPGPDAGAQDEAKEQRNGTKGLNDIFEAQKIEWHE
Targ1	M**HHHHHH**SSGVDLGTENLYFQSMASSLNEDPEGSRITYVKGDLFACPKTDSLAHCISEDCRMGAGIAVLFKKKFGGVQELLNQQKKSGEVAVLKRDGRYIYYLITKKRASHKPTYENLQKSLEAMKSHCLKNGVTDLSMPRIGCGLDRLQWENVSAMIEEVFEATDIKITVYTL
Mac1	MSYY**HHHHHH**LESTSLYKKAGFLEVLFQGPEVNSFSGYLKLTDNVYIKNADIVEEAKKVKPTVVVNAANVYLKHGGGVAGALNKATNNAMQVESDDYIATNGPLKVGGSCVLSGHNLAKHCLHVVGPNVNKGEDIQLLKSAYENFNQHEVLLAPLLSAGIFGADPIHSLRVCVDTVRTNVYLAVFDKNLYDKLVSSFLEMKSEK

bioRxiv preprint doi: https://doi.org/10.1101/2024.08.08.606661; this version posted August 9, 2024. The copyright holder for this preprint (which was not certified by peer review) is the author/funder, who has granted bioRxiv a license to display the preprint in perpetuity. It is made available under aCC-BY 4.0 International license.

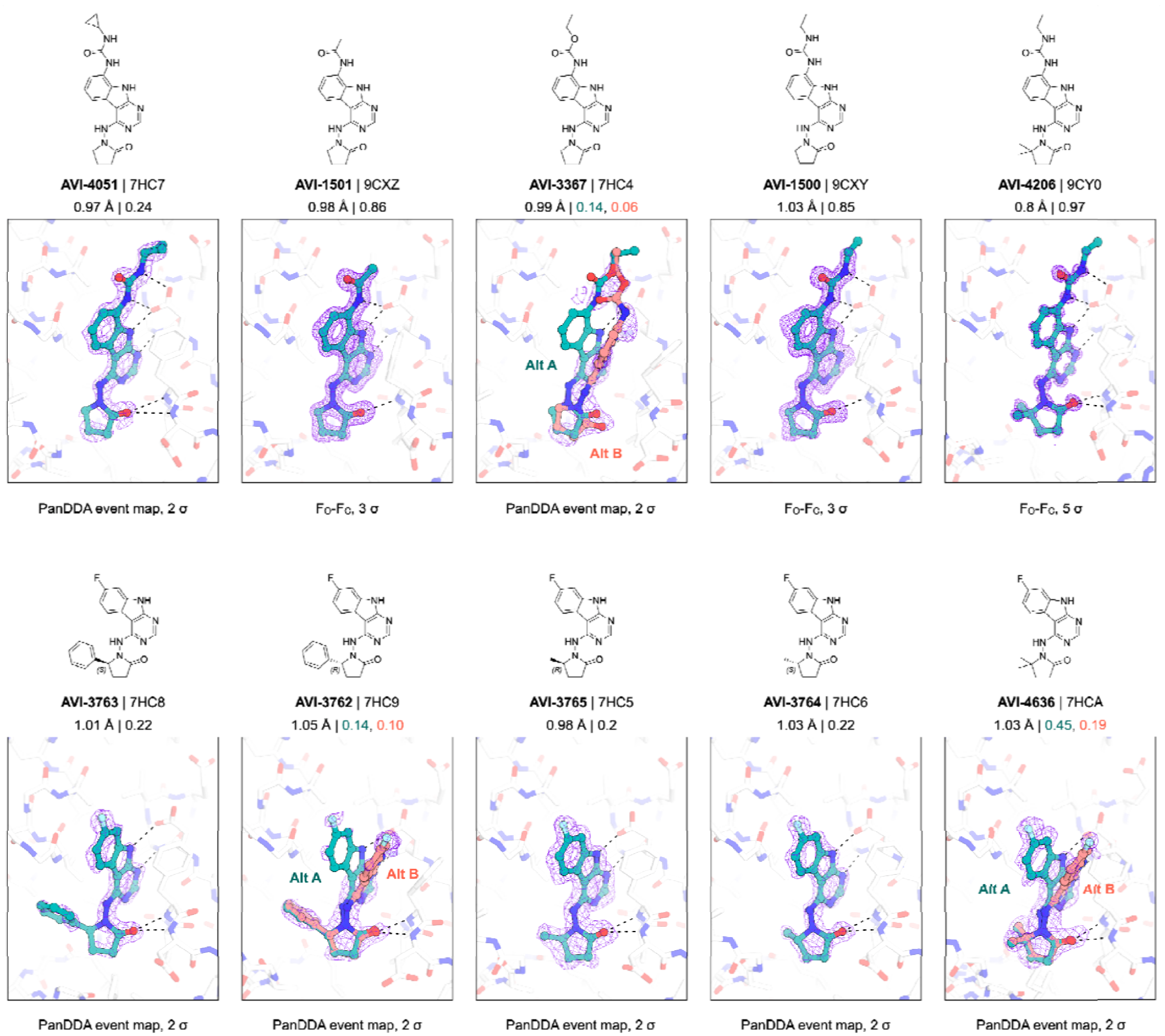

Supplementary Figure 1 - X-ray density for ligand modeling. Ligands were modeled using either traditional F_O-F_C electron density maps (AVI-1500, AVI-1501, AVI-4206) or PanDDA event maps (AVI-4051, AVI-3367, AVI-3763, AVI-3762, AVI-3765, AVI-3764 and AVI-4636). The diffraction resolution and refined occupancy are indicated for each ligand. The occupancy is indicated for each confirmation when multiple ligand poses were modeled.

bioRxiv preprint doi: https://doi.org/10.1101/2024.08.08.606661; this version posted August 9, 2024. The copyright holder for this preprint (which was not certified by peer review) is the author/funder, who has granted bioRxiv a license to display the preprint in perpetuity. It is made available under aCC-BY 4.0 International license.

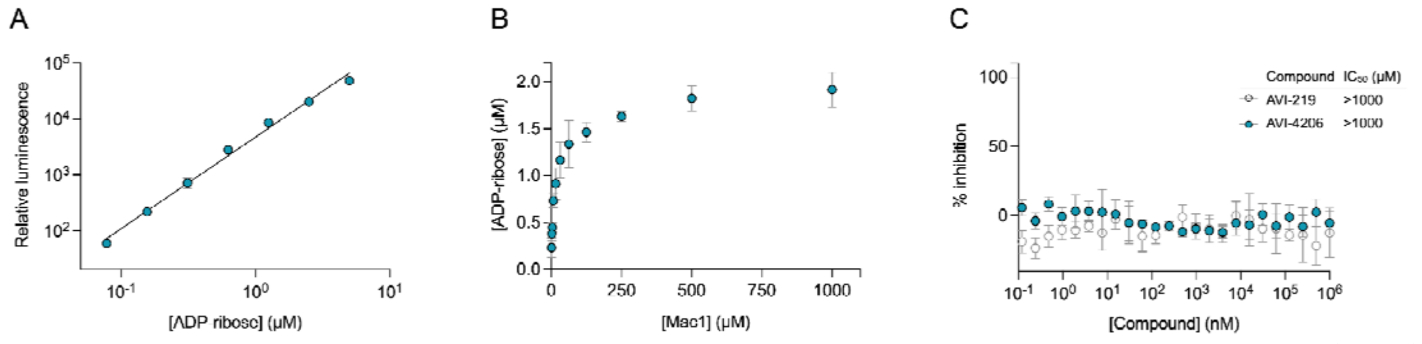

Supplementary Figure 2. AVI-4206 and AVI-219 inhibition of Mac1 determined using auto-mono-ADP-ribosylated PARP10 as a substrate.

(A) Standard curve of ADP-ribose detected using 100 nM NUDT5 and the AMP-Glo assay kit. Data are presented mean ± SD for four technical replicates. Data were fitted with a power function in the form $y = kx^a$ using non-linear regression (gray line).

(B) Titration of Mac1 with auto-mono-ADP-ribosylated PARP10. The concentration of PARP10 was 10 µM based on absorbance at 280 nm, but the titration indicated that the concentration of ADP-ribose released by Mac1 was five-fold lower (~2 µM). Data are presented mean ± SD for four technical replicates.

(C) Counterscreen of compounds against 100 nM NudT5 with 2 µM ADP-ribose as a substrate. No inhibition was detected up to 1 mM compound. Data are presented mean ± SD for four technical replicates.

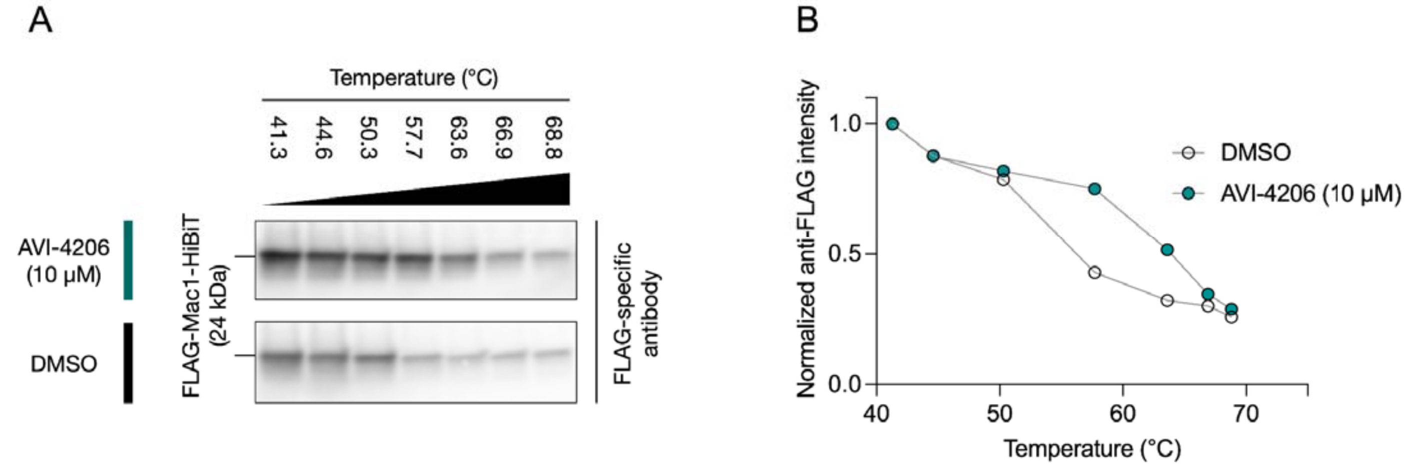

Supplementary Figure 3: AVI-4206 increases thermal stability of Mac1 in cells.

(A) CETSA-WB shows thermal stabilization of FLAG-tagged Mac1 protein after treatment of A549 cells with 10 µM of AVI-4206.

(B) Densitometry values were normalized to the lowest temperature for each treatment. Data are presented as a single densitometry measurement.

bioRxiv preprint doi: https://doi.org/10.1101/2024.08.08.606661; this version posted August 9, 2024. The copyright holder for this preprint (which was not certified by peer review) is the author/funder, who has granted bioRxiv a license to display the preprint in perpetuity. It is made available under aCC-BY 4.0 International license.

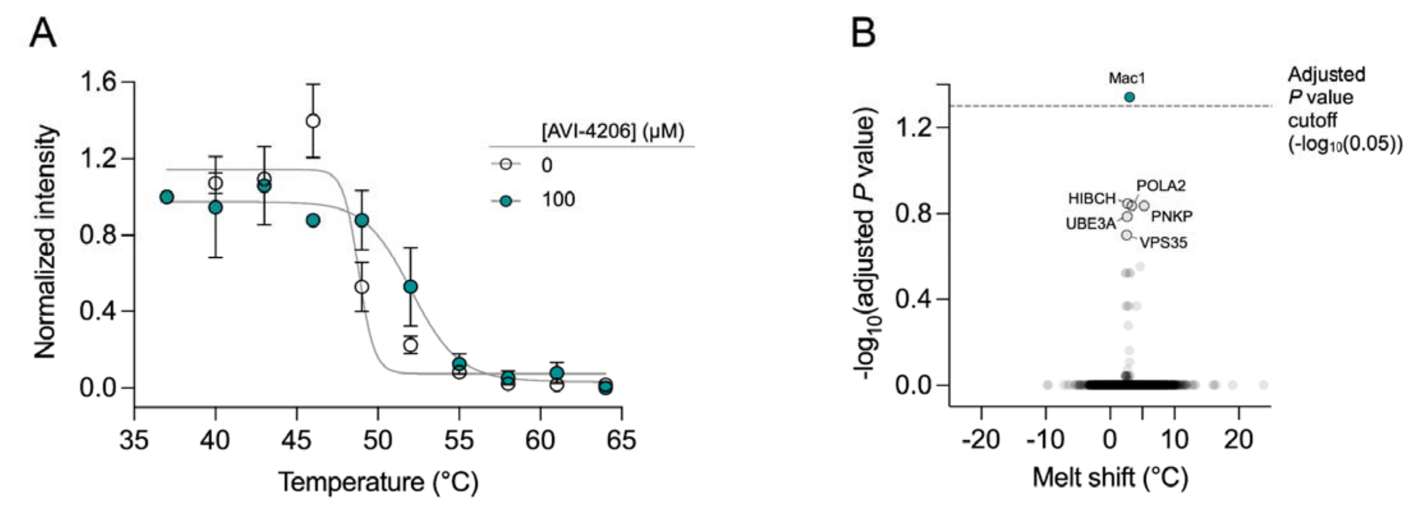

Supplementary Figure 4: **Thermal proteome profiling in A549 cellular lysates.**
(A) Melting curve for Mac1 in A549 lysates treated in duplicate with either DMSO or 100 µM of AVI-4206. Data were normalized to the mean intensity at 37°C. Data were fitted with a sigmoidal dose-response equation using non-linear regression (gray line).
(B) Volcano plot of the statistical significance and degree of melting temperature shift for all proteins with high quality melting curves (n= 3,446 proteins). Teal circles indicate proteins with a statistically significant shift in melting temperature (adjusted *P* value < 0.05). The highest non-significant proteins are labeled and do not have obvious functional overlap with macrodomains.

bioRxiv preprint doi: https://doi.org/10.1101/2024.08.08.606661; this version posted August 9, 2024. The copyright holder for this preprint (which was not certified by peer review) is the author/funder, who has granted bioRxiv a license to display the preprint in perpetuity. It is made available under aCC-BY 4.0 International license.

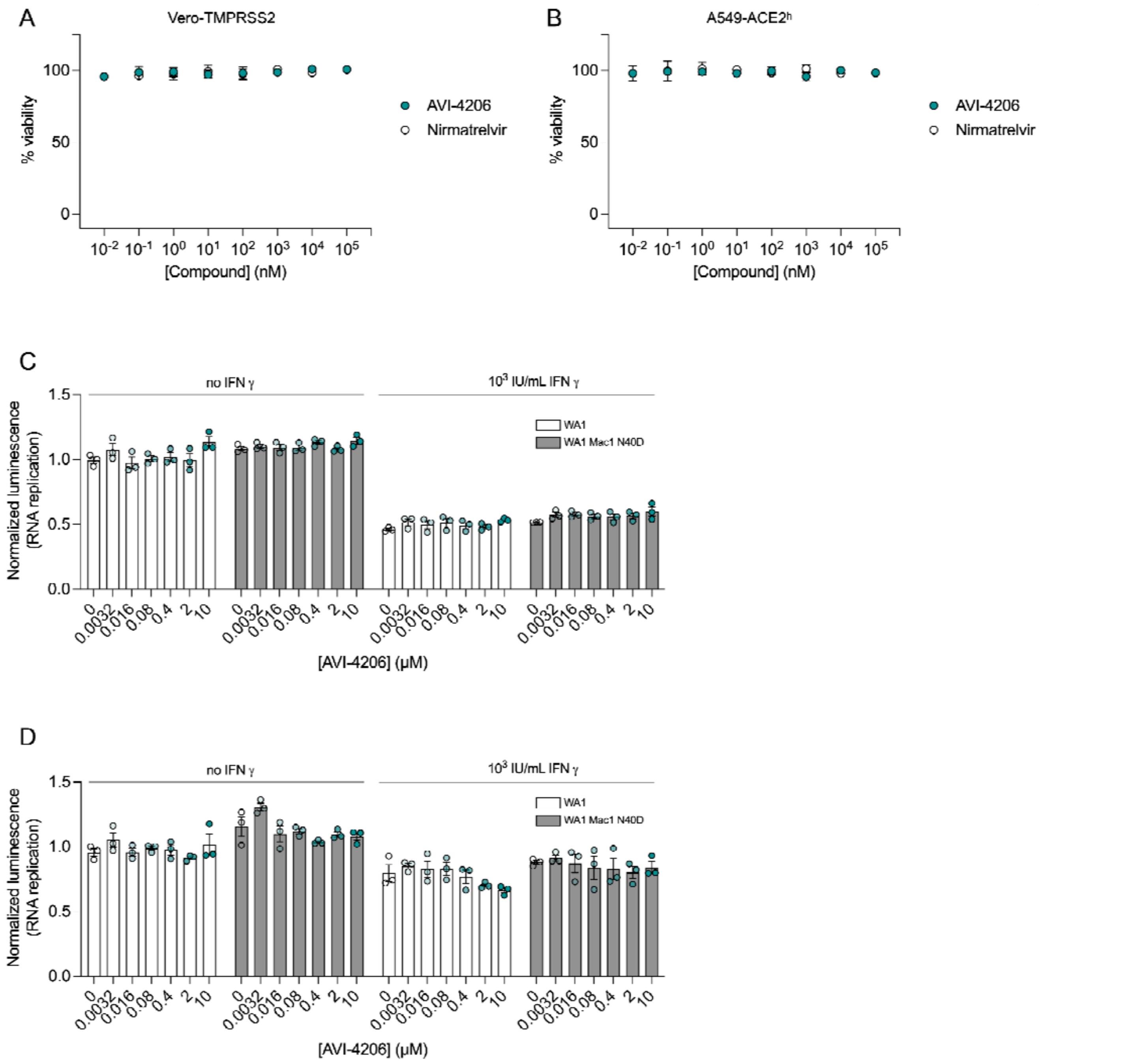

Supplementary Figure 5: AVI-4206 has limited antiviral efficacy and no cytotoxicity in cellular models of infection.

(A and B) Drug cytotoxicity of AVI-4206 in Vero-TMPRSS2 (A) and A549 ACE2^{h} (B) was measured using the CellTiter-Glo® viability assay. Graphs represent the mean ± SD of three biological replicates each conducted in triplicate.

(C and D) Luciferase readout of VAT (C) and A549 ACE2^{h} (D) cells infected with WA1 or WA1 Mac1 N40D replicons and treated with or without AVI-4206 and IFN-[illegible] at indicated concentrations. Results are plotted as normalized mean ± SD luciferase values of a representative biological experiment containing three technical replicates.

bioRxiv preprint doi: https://doi.org/10.1101/2024.08.08.606661; this version posted August 9, 2024. The copyright holder for this preprint (which was not certified by peer review) is the author/funder, who has granted bioRxiv a license to display the preprint in perpetuity. It is made available under aCC-BY 4.0 International license.

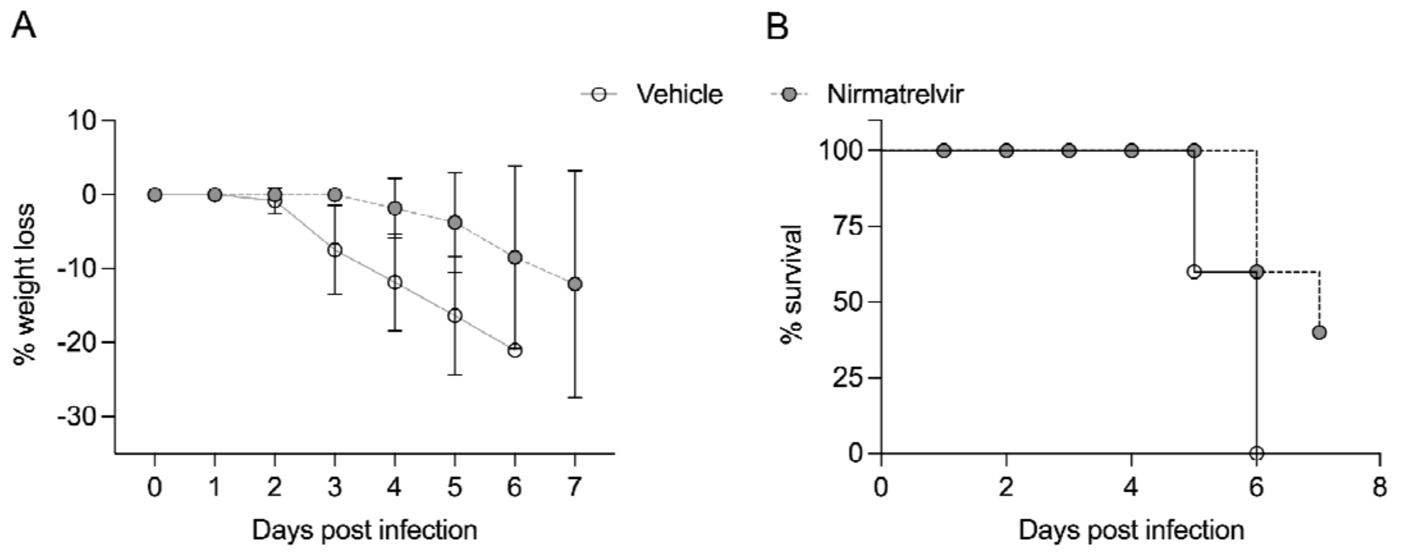

Supplementary Figure 6: Nirmatrelvir shows limited efficacy in the K18-hACE2 model of SARS-CoV-2 infection. **(A)** K18-hACE2 mice were infected with SARS-CoV-2 WA1 strain and dosed with nirmatrelvir (300 mg/kg) or vehicle BID. Each group was composed of n=5 mice. The percent weight loss is presented as mean ± SD.
(B) Survival curve in vehicle or nirmatrelvir treated mice based on the weight loss humane endpoint.

bioRxiv preprint doi: https://doi.org/10.1101/2024.08.08.606661; this version posted August 9, 2024. The copyright holder for this preprint (which was not certified by peer review) is the author/funder, who has granted bioRxiv a license to display the preprint in perpetuity. It is made available under aCC-BY 4.0 International license.

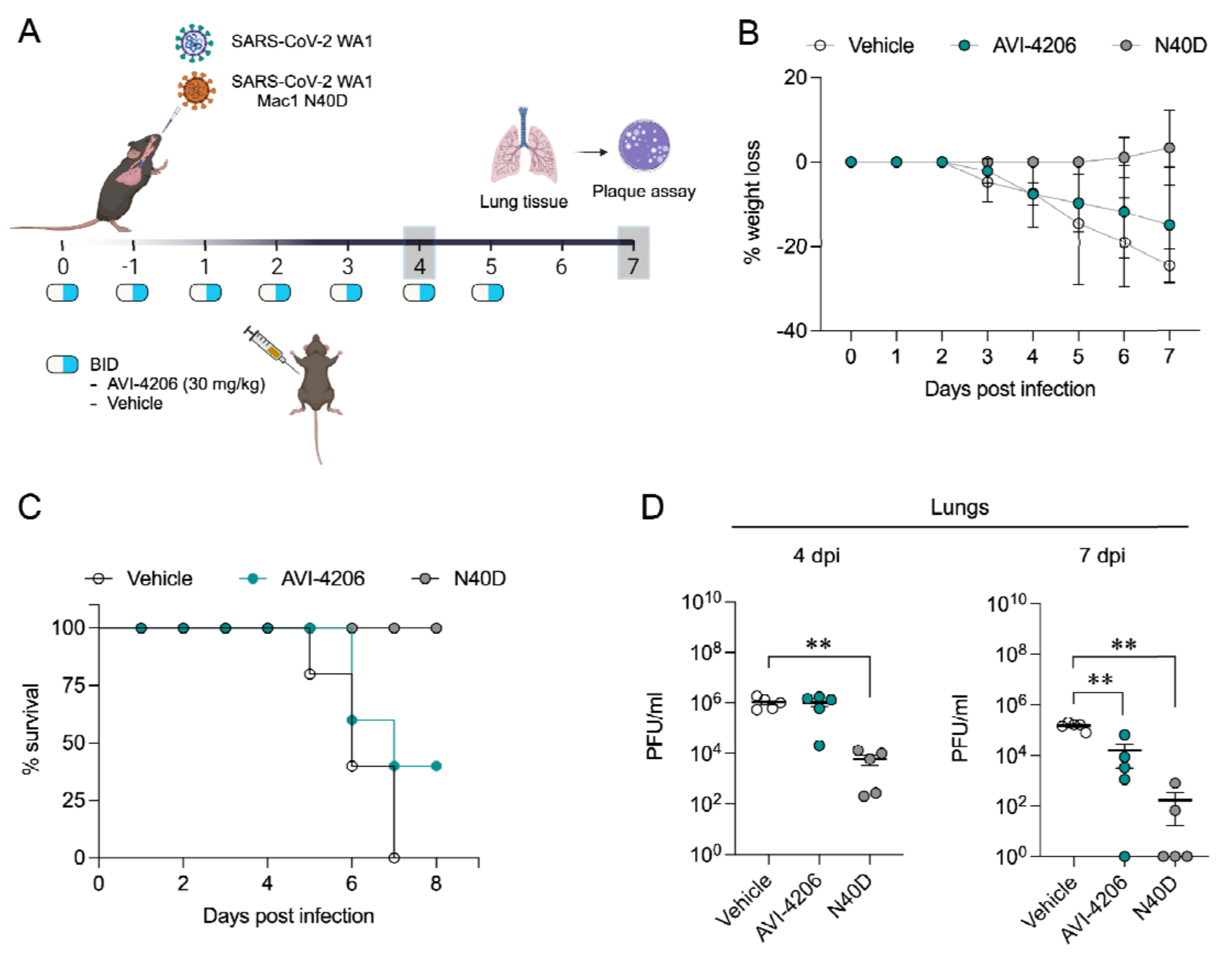

Supplementary Figure 7: **Lower dose AVI-4206 reduces viral replication and increases survival *in vivo***.
(A) K18-hACE2 mice were intranasally infected SARS-CoV-2 WA1 or SARS-CoV-2 WA1 Mac1 N40D mutant. Mice were treated as indicated with AVI-4206 (BID, 30 mg/kg) or vehicle. Each group was composed of n=10 mice (5 mice per time point).
(B) The percent body weight loss is presented as mean ± SD.
(C) Survival curve based on the percent body weight loss humane endpoint.
(D) Viral load in the lung at indicated time points is presented as mean ± s.e.m. **, $P < 0.01$ by Mann Whitney's test relative to the vehicle control.
